# The role of the bacterial protease Prc in the uropathogenesis of extraintestinal pathogenic *Escherichia coli*

**DOI:** 10.1186/s12929-019-0605-y

**Published:** 2020-01-03

**Authors:** Wen-Chun Huang, Chung-Yen Lin, Masayuki Hashimoto, Jiunn-Jong Wu, Ming-Cheng Wang, Wei-Hung Lin, Chang-Shi Chen, Ching-Hao Teng

**Affiliations:** 1 Institute of Molecular Medicine, College of Medicine, National Cheng Kung University, Tainan, Taiwan; 2 Institute of Basic Medical Sciences, College of Medicine, National Cheng Kung University, Tainan, Taiwan; 3 Institute of Information Science, Academia Sinica, Taipei, Taiwan; 4 Center of Infectious Disease and Signaling Research, National Cheng Kung University, Tainan, Taiwan; 5 Department of Biotechnology and Laboratory Science in Medicine, School of Biomedical Science and Engineering, National Yang Ming University, Taipei, Taiwan; 6 Department of Internal Medicine, National Cheng Kung University Hospital, College of Medicine, National Cheng Kung University, Tainan, Taiwan; 7 Department of Biochemistry and Molecular Biology, College of Medicine, National Cheng Kung University, Tainan, Taiwan

**Keywords:** Extraintestinal pathogenic *Escherichia coli*, Urinary tract infections, Protease Prc, Motility, Flagella, σ^E^, Two-component signal transduction system RcsCDB, Spr

## Abstract

**Background:**

Extraintestinal pathogenic *E. coli* (ExPEC) remains one of the most prevalent bacterial pathogens that cause extraintestinal infections, including neonatal meningitis, septicemia, and urinary tract (UT) infections (UTIs). Antibiotic therapy has been the conventional treatment for such infections, but its efficacy has decreased due to the emergence of antibiotic-resistant bacteria. Identification and characterization of bacterial factors that contribute to the severity of infection would facilitate the development of novel therapeutic strategies. The ExPEC periplasmic protease Prc contributes to the pathogen’s ability to evade complement-mediated killing in the serum. Here, we further investigated the role of the Prc protease in ExPEC-induced UTIs and the underlying mechanism.

**Methods:**

The uropathogenic role of Prc was determined in a mouse model of UTIs. Using global quantitative proteomic analyses, we revealed that the expression of FliC and other outer membrane-associated proteins was altered by Prc deficiency. Comparative transcriptome analyses identified that Prc deficiency affected expression of the flagellar regulon and genes that are regulated by five extracytoplasmic signaling systems.

**Results:**

A mutant ExPEC with a *prc* deletion was attenuated in bladder and kidney colonization. Global quantitative proteomic analyses of the *prc* mutant and wild-type ExPEC strains revealed significantly reduced flagellum expression in the absence of Prc, consequently impairing bacterial motility. The *prc* deletion triggered downregulation of the flhDC operon encoding the master transcriptional regulator of flagellum biogenesis. Overexpressing *flhDC* restored the *prc* mutant’s motility and ability to colonize the UT, suggesting that the impaired motility is responsible for attenuated UT colonization of the mutant. Further comparative transcriptome analyses revealed that Prc deficiency activated the σ^E^ and RcsCDB signaling pathways. These pathways were responsible for the diminished *flhDC* expression. Finally, the activation of the RcsCDB system was attributed to the intracellular accumulation of a known Prc substrate Spr in the *prc* mutant. Spr is a peptidoglycan hydrolase and its accumulation destabilizes the bacterial envelope.

**Conclusions:**

We demonstrated for the first time that Prc is essential for full ExPEC virulence in UTIs. Our results collectively support the idea that Prc is essential for bacterial envelope integrity, thus explaining how Prc deficiency results in an attenuated ExPEC.

## Background

Extraintestinal pathogenic *Escherichia coli* (ExPEC) is one of the most common bacterial pathogens causing bacteremia, neonatal meningitis, and urinary tract (UT) infections (UTIs) [1]. The diseases caused by ExPEC have resulted in substantial morbidity, mortality, and healthcare costs [1, 2]. Antibiotic therapy is the traditional way to treat *E. coli* infections. However, the rapid emergence of antibiotic-resistant strains has become a serious problem in managing bacterial infections because of the shortage of novel and effective antibiotics [3]. Accordingly, new antimicrobial strategies against *E. coli*-associated infections are urgently needed. As bacterial factors required for maintaining the virulence of ExPEC are potential antimicrobial targets, identifying such factors and understanding how they contribute to infections would facilitate the development of novel treatment strategies.

The *E. coli* periplasmic protease Prc is required for ExPEC to cause a high level of bacteremia [4] since ExPEC lacking Prc displays enhanced sensitive to complement-mediated serum killing and thus is defective in survival in the host bloodstream [4]. In addition to that in ExPEC, Prc homologs in other pathogenic bacteria have also been shown to contribute to bacterial pathogenesis. For example, a *prc* mutant of *Salmonella typhimurium* exhibits a diminished ability to survive in murine macrophages and attenuated virulence in mice [5]. Disruption of the Prc-homologous protein CtpA in the animal pathogens *Brucella suis* and *Burkholderia mallei* decreases the abilities of these bacteria to survive in murine macrophages [6, 7]. Mutation of *prc* in the plant pathogen genus *Xanthomonas* results in decreased virulence, biofilm production, and resistance to environmental stresses [8, 9]. In this study, we further demonstrate the novel pathogenic role of Prc in ExPEC UTIs.

The mechanism of how the Prc protease and its homologs contribute to bacterial virulence remains to be elucidated. Our previous study has shown that deletion of *prc* in the ExPEC strain RS218, which is associated with neonatal meningitis, significantly changed the protein profiles in the outer membrane (OM) fraction [4]. The altered protein expression in the OM fraction may contribute to the defective ability to cause infections since OM-associated proteins (OMPs) are the major factors involved in bacterium-host interactions and play key roles in maintaining the integrity of the OM, which is the main bacterial structure for sensing and coping with the harsh host environment during infections [10].

Flagella are the protein structures associated with the bacterial OM that mediate bacterial motility [11]. Flagella of ExPEC have been shown to contribute to the pathogenesis of UTIs because these structures enable the bacteria to disseminate, and they facilitate colonization and ascension of the UT [12–15]. The process of flagellum biogenesis is regulated by the flagellar regulon organized in a three-tier hierarchy [16]. Three flagellar genes, *flhD*, *flhC*, and *fliA*, are central for the hierarchical expression of this regulon. At the top of this hierarchy (class 1) are the master operon genes, *flhDC*. Their gene products, FlhD and FlhC, assemble into a heterohexamer (FlhD_4_C_2_) [17] that acts as an essential transcription activator of the class 2 genes. Class 2 genes encode the flagellum-specific sigma factor σ^28^ (FliA), the flagellar basal body and hook proteins, etc. Class 3 genes encode the subunit of the flagellar filament (FliC), stator components of the flagellar motor, as well as the chemotaxis pathway.

The flagellar regulon is highly regulated by environmental cues, such as osmolality, nutrients, cell density, and temperature [18]. *E. coli* cells perceive and respond to such external environmental stimuli through extracytoplasmic stress signaling systems (ESSSs) whose activation triggers transcriptional reprogramming, allowing the bacteria to cope with the corresponding external conditions. Two-component signal transduction systems (2CSTSs) are among the members of ESSSs. The activation of some 2CSTSs, including RcsCDB, CpxA-CpxR, EnvZ-OmpR, and QseB-QseC, has been shown to suppress the expression of the flagellar regulon, [19–24]. In *E. coli*, the prototypical 2CSTSs consist of an inner membrane-bound sensor kinase and a DNA-binging cytoplasmic response regulator. In response to specific stimuli, the sensor kinase is autophosphorylated at a conserved histidine residue. Then, the phosphoryl group is transferred to a conserved aspartate in the cognate response regulator. Finally, the phosphorylated response regulator up- or downregulates the transcription of target genes to induce cellular responses to external signals. In addition to 2CSTSs, the alternative sigma factor σ^E^ governs an extracytoplasmic signaling pathway that responds to heat-shock stress [25, 26]. Although no study has demonstrated whether the activation of this heat-shock response system suppresses flagellum expression, it is known that bacterial motility is suppressed under high environmental temperatures, suggesting that the σ^E^ system may contribute to the regulation of the flagellar regulon [27]. In unstressed bacteria, σ^E^ is sequestered in the cytoplasmic side of the inner membrane by the antisigma factor RseA, which is an inner membrane-spanning protein with a periplasmic-exposed C-terminus and a cytoplasmic-exposed N-terminus [28–30]. Under stress, RseA is proteolytically degraded by the sequential action of the periplasmic and cytoplasmic proteases DegS and RseP, resulting in release of σ^E^ into the cytoplasm, in which this sigma factor can associate with the core enzyme of RNA polymerase to allow σ^E^-regulated gene transcription [31–36]. The *E. coli* envelope, which is composed of the OM, inner membrane (IM), periplasm, and peptidoglycan mesh [37] is the frontline of bacterial interaction with the external environment. Alteration of the envelope components has been shown to be able to activate ESSSs, similar to environmental stresses. It remains unclear whether the altered protein profile of the OM fraction caused by Prc deficiency could activate the signaling systems.

As a periplasmic protease, Prc exerts its biological function through proteolytic regulation of its substrates, which are supposed to be located in or partially exposed to the periplasmic space. The attenuated virulence resulting from Prc deficiency in bacterial pathogens may be a consequence of the substrate dysregulation caused by the loss of proteolytic control. In *E. coli*, the periplasm-exposed OM protein Spr has been shown to be a substrate of the Prc protease. Spr is a peptidoglycan hydrolase. Singh et al. have shown that deletion of *prc* causes Spr accumulation in *E. coli* cells [38]. The protein accumulation contributes to the mutant’s growth defect under low osmolarity at 42 °C [38].

In this study, we found that the Prc protease of ExPEC is necessary for maintaining intact bacterial motility that is important for UT colonization. The underlying mechanism was shown to be involved in the activation of ESSSs and the intracellular level of the Prc substrate Spr.

## Methods

### Bacterial strains, plasmids and growth conditions

The bacterial strains and plasmids used in this study are shown in Table 1. Bacteria were grown in Luria Bertani (LB) broth containing 1% tryptone, 0.5% NaCl and 1% yeast extract at 37 °C overnight (approximately 16 h) and were stored in LB with a final concentration of 15% glycerol at − 80 °C.

**Table 1:** *E. coli* strains and plasmids used in this study

Strains or plasmids	Relevant information	Reference
Strains		
WT-RS218	RS218 isolated from the cerebrospinal fluid of a neonate with meningitis	[4]
Δ*prc*-RS218	RS218 with a *prc* deletion	[4]
Δ*lacZ*-RS218	RS218 with a *lacZ* deletion	This study
Δ*lacZ*Δ*prc*-RS218	RS218 with *lacZ* and *prc* deletions	This study
Δ*rseA*-RS218	RS218 with a *rseA* deletion	This study
Δ*degS*Δ*prc*-RS218	RS218 with *degS* and *prc* deletions	This study
*lacZ*::*degS*Δ*degS*Δ*prc*-RS218	Δ*degS*Δ*prc*-RS218 with complementary *degS* at the *lacZ* gene chromosomal locus	This study
Δ*rcsB*Δ*prc*-RS218	RS218 with *rcsB* and *prc* deletions	This study
*lacZ*::*rcsB*Δ*rcsB*Δ*prc*-RS218	Δ*rcsB*Δ*prc*-RS218 with complementary *rcsB* at the *lacZ* gene chromosomal locus	This study
Δ*degS*Δ*rcsB*Δ*prc*-RS218	RS218 with *degS*, *rcsB* and *prc* deletions	This study
Δ*ompR*Δ*prc*-RS218	RS218 with *ompR* and *prc* deletions	This study
Δ*qseB*Δ*prc*-RS218	RS218 with *qseB* and *prc* deletions	This study
Δ*cpxR*Δ*prc*-RS218	RS218 with *cpxR* and *prc* deletions	This study
Spr-3xFlag-RS218	RS218 with *spr*-3xFlag tag at the *spr* gene chromosomal locus	This study
Spr-3xFlag-Δ*prc*-RS218	RS218 with *spr*-3xFlag tag and *prc* deletion at the *spr* gene chromosomal locus	This study
Δ*spr*Δ*prc*-RS218	RS218 with *spr* and *prc* deletions	This study
Δ*fliC*-RS218	RS218 with a *fliC* deletion	This study
WT-CFT073	CFT073 isolated from the blood and urine of a woman with acute pyelonephritis	[39]
Δ*prc*-CFT073	CFT073 with a *prc* deletion	This study
Δ*lacZ*-CFT073	CFT073 with a *lacZ* deletion	This study
Δ*lacZ*Δ*prc*-CFT073	CFT073 with *lacZ* and *prc* deletions	This study
Δ*degS*Δ*prc*-CFT073	CFT073 with *degS* and *prc* deletions	This study
Δ*rcsB*Δ*prc*-CFT073	CFT073 with *rcsB* and *prc* deletions	This study
Δ*fliC*-CFT073	CFT073 with a *fliC* deletion	This study
WT-UTI89	UTI89 isolated from the urine of a patient with cystitis	[40]
Δ*prc*-UTI89	UTI89 with a *prc* deletion	This study
Δ*lacZ*-UTI89	UTI89 with a *lacZ* deletion	This study
Δ*lacZ*Δ*prc*-UTI89	UTI89 with *lacZ* and *prc* deletions	This study
Δ*fliC*-UTI89	UTI89 with a *fliC* deletion	This study
Plasmids		
pCL1920	Low copy-number plasmid	[41]
pPrc	pCL1920 harboring the *prc* gene that is under control of the *lac* promoter	[4]
pPrc-S430A	pCL1920 harboring the *prc* gene containing a serine point mutation resulting in inactivation of the serine protease	This study
pPrc-K455A	pCL1920 harboring the *prc* gene containing a lysine point mutation resulting in inactivation of the serine protease	This study
pUC19	Expression plasmid containing the *lac* promoter	NEB
pFlhDC	pUC19 harboring an HA-tagged *flhD* and a His-tagged *flhC gene* that are under control of the *lac* promoter	This study
pACYC184	Expression plasmid containing constitutively expressed Tet^r^ and Cm^r^ genes	NEB
pDegQ	pACYC184 harboring the *degQ* gene that is under control of chloramphenicol resistant gene’s promoter	This study
pBAD	Expression plasmid containing an arabinose-inducible promoter	Invitrogen
pBAD-FlhDC	pBAD harboring a HA-tagged *flhD* and a His-tagged *flhC* gene that are under control of an arabinose-inducible promoter	This study
pRcsB	pBAD harboring the *rcsB* gene that is under control of an arabinose-inducible promoter	This study
pRseA	pBAD harboring the HA-tagged-*rseA gene*-His-tagged that is under control of an arabinose-inducible promoter	This study
pSpr	pBAD harboring a Flag-tagged *spr* gene that is under control of an arabinose-inducible promoter	This study

### Construction of mutants and plasmids

The ExPEC mutants were constructed using polymerase chain reaction (PCR) product-based λ Red recombination, as described previously [42]. The plasmids pCA3 × Flag [43] and pKD3 [42] served as templates for synthesizing the 3 × Flag- and chloramphenicol resistance cassette-encoding sequences by PCR, respectively. The primers used for mutant construction are shown in Additional file 1: Table S1.

The plasmids producing the Prc-S430A or Prc-K455A mutation proteins were generated by site-directed mutagenesis using the corresponding primer (Additional file 1: Table S1). The complementary and overexpression plasmids newly constructed in the study were created by cloning the indicated PCR-amplified gene fragments into the corresponding plasmid vectors. The primers for amplification of the gene fragments are shown at Additional file 1: Table S1.

### Mouse model of urinary tract infection (UTI)

The animal UTI studies were performed as described previously [12], with some modification. For each experiment, 2 ExPEC strains were mixed at a ratio of 1:1. Eight-week-old female C3H/HeN mice were anesthetized and transurethrally inoculated with a 50-μl bacterial suspension (1 × 10^8^ colony-forming unit, CFU) per mouse using a sterile polyethylene catheter connected to an infusion pump (Harvard Apparatus, Holliston, MA, USA) with a flow rate of 100 μl/min. Subsequently, 48 h post-infection, mice were sacrificed, and their bladders and kidneys were collected, weighed, and homogenized in sterile culture tubes containing 3 ml of normal saline. Bacterial counts were differentiated and determined by plating the homogenates onto LB agar plates containing IPTG and X-gal. The strains with and without *lacZ* showed blue and white colonies on the plates, respectively.

### Liquid chromatography-tandem mass spectrometry (LC/MS/MS) analysis of bacterial proteins

To prepare bacterial proteins for LC/MS/MS analysis, 16-h cultures of WT-RS218 and Δ*prc*-RS218 (three independent cultures for each strain) were harvested and subjected to French press at 8000 lb./in^2^. The resulting bacterial lysates were subjected to 12.5% SDS-PAGE to separate the proteins in the samples. The gel lane of each sample was cut into 5 slices. The gel slices were subjected to in-gel digestion with trypsin, followed by protein identification with the Thermo LTQ-Orbitrap Velos system. The MS/MS spectra were searched against *Escherichia coli* SwissProt 2014_08 (546,238 sequences; 194,363,168 residues) using Sequest (version 27, rev 12), which is part of the BioWorks 3.3 data analysis package (Thermo Fisher, San Jose, CA, USA). Subsequently, protein identifications with 2 peptides in at least one of the samples were retained. The proteins that are defined to be located on or associated with the outer membrane (OM) based on the EcoCyc database (http://ecocyc.org/), showed at least a 2-fold change with statistical significance between WT-RS218 and Δ*prc*-RS218 were identified (Table 2).

**Table 2:** Identification of altered OMPs by liquid chromatography-tandem mass spectrometry

Protein name	Description	Fold change^a^	*P* value
Downregulated protein in Δ*prc*-RS218			
FliC	Flagellin	(−) 100	2.50E-04
SlyB	Outer membrane lipoprotein SlyB	(−) 100	5.30E-04
Upregulated protein in Δ*prc*-RS218			
Spr	Murein DD-endopeptidase	(+) 100	1.10E-03
BamA	Outer membrane protein assembly factor	(+) 100	3.30E-04
Ag43	Antigen 43	(+) 100	7.80E-04
Tsx	Nucleoside-specific channel-forming protein	(+) 100	9.78E-07
TraT	Conjugal transfer surface exclusion protein TraT	(+) 3.17	1.55E-02
TolC	Outer membrane channel protein TolC	(+) 2.21	4.47E-03

^a^(−), indicates that the protein was downregulated in Δ*prc*-RS218 compared to in WT-RS218

(+), indicates that the protein was upregulated in Δ*prc*-RS218 compared to in WT-RS218

### Western blot analysis

The protein levels in the OM fractions or bacterial lysates were determined by western blot analyses. Preparation of the protein samples was performed as described previously [4]. The primary antibodies utilized to detect FliC, Prc, and OmpA were rabbit antisera against FliC (anti-H7, Becton Dickinson, Sparks, MD, USA) and Prc, and a mouse anti-OmpA antiserum. The recombinant proteins fused with HA and Flag tags were detected with a mouse anti-HA antibody and rabbit anti-Flag antibody, respectively (Sigma-Aldrich, St. Louis, MO, USA).

### Motility assay

Bacterial strains were stab inoculated onto 0.3% agar plates and incubated at 37 °C for 10 h [44]. The diameter of motility was measured and is shown in the quantified figure.

### RNA isolation

Total RNA was extracted from 16-h cultures of bacteria using the RNeasy Mini kit (QIAGEN, Hilden, Germany) according to the manufacturer’s instructions. To remove contaminating DNA, the resulting RNA was incubated with DNase I (Roche Applied Science, Mannheim, Germany) at 37 °C for 1.5 h. Then, the mixture was subjected to phenol / chloroform (1:1) (Sigma-Aldrich, St. Louis, MO, USA) extraction and ethanol precipitation. Finally, the purified RNA was dissolved in RNase-free water and stored at − 80 °C.

### RNA sequencing (RNA-seq) and identification of differentially expressed genes

The total RNA samples from the WT-RS218 and Δ*prc*-RS218 16-h cultures (three independent culture samples for each strain) were subjected to cDNA library construction (paired-end) using a TruSeq RNA sample preparation kit (Illumina, San Diego, CA, USA). Sequencing was performed with the Illumina Solexa platform according to the manufacturer’s protocol (Illumina, San Diego, CA, USA). Qualified reads were aligned to the reference genome of the ExPEC strain UTI89 because the genomes of RS218 and UTI89 are very similar [40, 45]. The transcriptional level of gene expression was calculated and normalized by fragments per kilobase of transcript per million mapped reads (FPKM) [46]. The differentially expressed genes between the wild-type strain and the *prc* mutant (*P* value < 0.05) were analyzed by Regulatory Network Interactions of RegulonDB database [47] and classified into different regulons (Table 3).

**Table 3:** The differentially expressed flagellum- and five ESSSs-related genes between WT-RS218 and Δ*prc*-RS218 by RNA-seq analysis

Gene name	Gene product / functional description	Fold change^a^	*P* value
Flagellum-related genes			
*flhD*	Transcriptional activator of flagellar class II operons	0.73	0.019
*fliT*	Chaperone of flagellar export system	0.59	0.002
*fliP*	Flagellin export apparatus, integral membrane protein	0.51	0.004
*fliE*	Flagellar synthesis; basal body component	0.46	0.003
*fliD*	Hook-associated protein 2	0.31	0.011
σ^E^ regulon			
*wza*	Polysaccharide export protein	9.12	0.015
*wzc*	Tyrosine-protein kinase Etk/Wzc	3.49	0.036
*hpf*	Putative σ^N^ modulation protein	2.66	0.041
*uspD*	Universal stress protein D	2.50	0.025
*rpoN*	RNA polymerase subunit, σ^N^	2.46	0.016
*yiiS*	Unknown function	2.42	0.033
*iaaA*	Beta-aspartyl-peptidase	2.40	0.001
*ecfJ*	Unknown function	2.07	0.002
*yeaY*	Predicted lipoprotein	2.02	0.029
*insK*	IS150 conserved protein Insb, integrase core domain protein	1.84	0.018
*fabZ*	3R-hydroxymyristoyl acyl carrier protein (ACP) dehydratase	1.79	0.008
*bamE*	Lipoprotein stabilizer of BamABCDE OM biogenesis complex	1.74	0.033
*rseA*	Anti-RNA polymerase sigma factor σ^E^	1.53	0.013
*tolB*	Tol-Pal cell envelope complex	1.44	0.025
*clpX*	ATPase subunit of ClpXP protease	1.43	0.034
*rutR*	TetR/AcrR family transcriptional regulator	1.41	0.029
*mdoG*	Periplasmic glucan biosynthesis	1.37	0.006
*lptD*	LPS assembly outer membrane complex protein	1.35	0.041
*plsB*	Glycerol-3-phosphate O-acyltransferase	1.34	0.027
*rfaD*	ADP-L-glycero-D-mannoheptose-6-epimerase	1.33	0.003
*fkpA*	Periplasmic peptidylprolyl cis-trans isomerase	1.23	0.004
RcsB regulon			
*yjbE*	Exopolysaccharide production protein	12.37	0.035
*wza*	Polysaccharide export protein	9.12	0.015
*wzc*	Tyrosine-protein kinase Etk/Wzc	3.49	0.036
*osmC*	Osmotically inducible protein OsmC	2.32	0.035
*osmB*	Osmotically inducible lipoprotein	1.90	0.048
*rarA*	Recombinase RarA	1.39	0.006
CpxR regulon			
*mviM*	Putative virulence factor	2.09	0.016
*aroG*	2-dehydro-3-deoxyphosphoheptonate aldolase	1.93	0.004
*mdtC*	MdtABC-TolC efflux pump	1.52	0.026
*slt*	Soluble lytic murein transglycosylase	1.49	0.02
*ppiA*	Peptidylprolyl cis-trans isomerase A	1.16	0.012
*mdtB*	MdtABC-TolC efflux pump	1.15	0.002
QseB regulon			
*qseB*	Quorum sensing two-component response regulator	1.50	0.018
OmpR regulon			
*ompC*	Outer membrane porin protein C	3.29	0.031

^a^, indicates fold change relative to WT-RS218

### Real-time quantitative PCR (qPCR) and reverse transcription-PCR (RT-PCR)

The purified RNA was reverse transcribed into cDNA by using random hexamer primers and Moloney murine leukemia virus (M-MLV) reverse transcriptase according to the manufacturer’s instructions (Invitrogen, Carlsbad, CA, USA). For qPCR, the cDNA and primers were mixed with KAPA SYBR FAST qPCR Master Mix (Kapa Biosystems, Boston, MA, USA) and then subjected to PCR using a StepOnePlus™ Real-Time PCR System (Applied Biosystems, Carlsbad, CA, USA). The expression levels of the genes were normalized to those of *ftsZ*. For RT-PCR, the cDNA was subjected to PCR amplification using Taq polymerase for 25 cycles. The resulting products were analyzed by gel electrophoresis and visualized by ethidium bromide (EtBr) staining. The primers used for these assays are shown in Additional file 1: Table S1.

### Statistical analysis

Animal UTI experiments were analyzed by using a nonparametric Wilcoxon matched-pair test. The statistical significance of the other experiments were analyzed by unpaired two-tailed Student’s *t* test. A *P* value of < 0.05 was considered statistically significant. *, *P* < 0.05; **, *P* < 0.01; ***, *P* < 0.001; and ****, *P* < 0.0001. (GraphPad Prism 7; GraphPad Software Inc., La Jolla, CA, USA).

## Results

### Prc deficiency decreases the ability of extraintestinal pathogenic *Escherichia coli* (ExPEC) to cause urinary tract (UT) infections (UTIs)

**Fig. 1:**
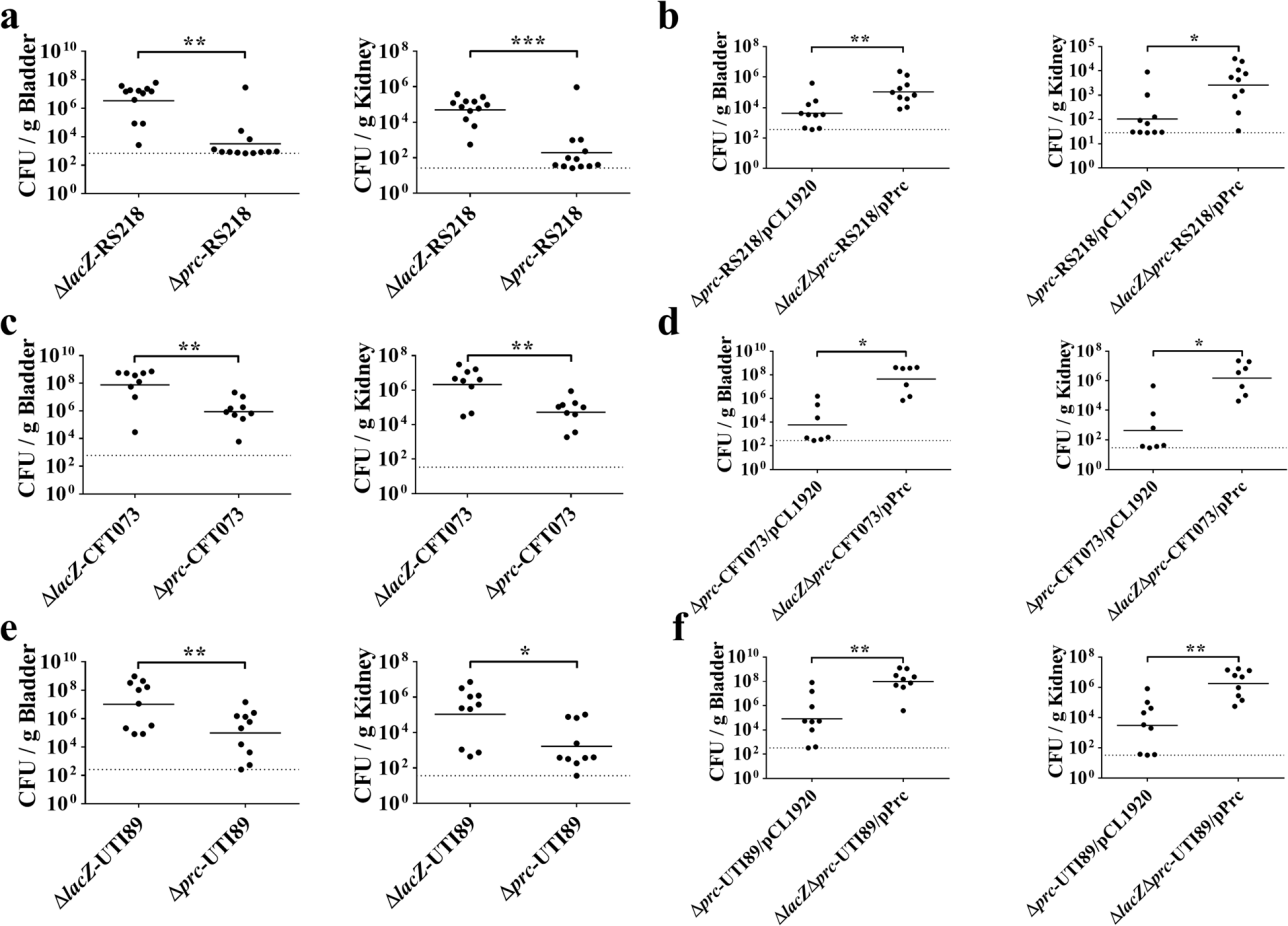
Transurethral cochallenge of mice with the ExPEC strains with or with the *prc* mutation. (**a**, **c**, and **e**) Cochallenges of mice with ExPEC *prc* mutants and their otherwise wild-type strains. Equal numbers of the *prc* mutants of the indicated ExPEC strains and their otherwise wild-type strains (the *lacZ* mutants) were transurethrally inoculated into mice. At 48 h post-inoculation (hpi), bacterial colonization levels of the bladder and kidney were determined. (**b**, **d**, and **f**) Cochallenges of mice with the indicated ExPEC *prc* mutants and their corresponding complemented strains. *lacZ* and *prc* double deletion mutants (∆*lacZ*∆*prc*) harboring the *prc*-encoding plasmid pPrc (Table 1) served as the complemented strains. Equal numbers of the *prc* mutants harboring the empty vector pCL1920 and the corresponding complemented strains were transurethrally coinoculated into mice. The bacterial counts of each strain in the bladders and kidneys were determined at 48 hpi. The bacterial counts of the mutants and the otherwise wild-type or complemented strains in the same organs were differentiated and enumerated by spreading the homogenized infected tissue onto LB agar plates with IPTG and X-gal. Horizontal bars indicate the median level of the bacterial counts. The dotted line represents the limit of detection

We have previously shown that Prc is required for ExPEC to cause a high level of bacteremia [4]. Since ExPEC is the most common cause of UTIs, in the present study, we further investigated whether Prc contributes to ExPEC UTIs. The ability of a *prc* mutant of the ExPEC strain RS218 (Δ*prc*-RS218) to colonize the UT was compared to that of an otherwise wild-type *lacZ* mutant (Δ*lacZ*-RS218). Deletion of *lacZ* did not affect the ability of ExPEC to colonize the UT (data not shown). After transurethrally infecting a 1:1 mixture of the two bacteria into mice for 48 h, the bacterial loads in the bladder and kidney were determined. Δ*prc*-RS218 showed significantly lower bacterial counts than the otherwise wild-type strain in the bladder and kidney (Fig. 1a). In addition, trans-complementation with the *prc* gene significantly restored the ability to colonize the organs (Fig. 1b). Similarly, *prc* mutants of the ExPEC strains CFT073 and UTI89, which were isolated from patients with UTIs, showed decreased abilities to colonize the UT, and trans-complementation of the mutants with the *prc* gene restored their bacterial counts in the organs (Fig. 1c, d, e, and f). These results demonstrated that Prc contributes to the pathogenesis of UTIs.

### Prc deficiency alters the expression of outer membrane (OM)-associated proteins (OMPs) in ExPEC

**Fig. 2:**
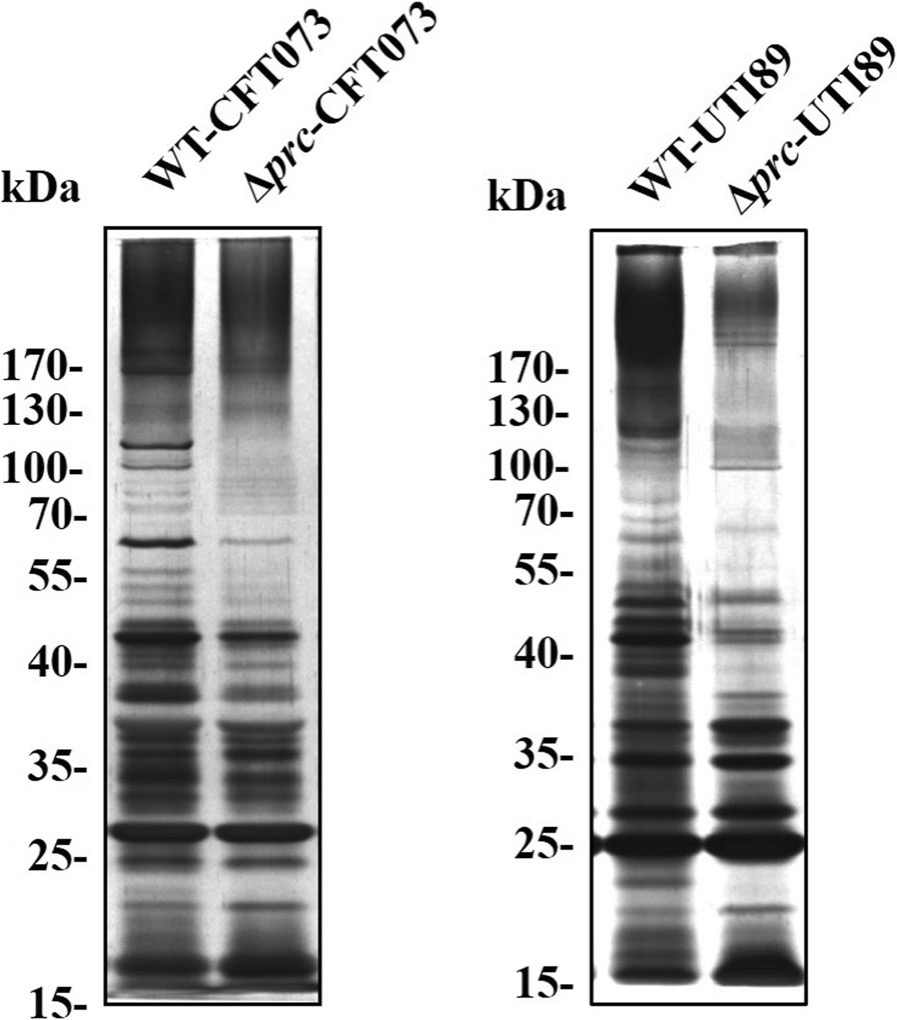
The OMP profiles of the ExPEC strains CFT073 and UTI89 and their *prc* mutants. The outer membrane proteins of the indicated bacteria were separated by 10% SDS-PAGE and then subjected to silver staining

It has been demonstrated that deletion of *prc* alters the OMP profile in RS218 [4]. As shown in Fig. 2, *prc* deletion changed the OMP profiles of CFT073 and UTI89 as well. These findings suggested that alteration of OMP expression is a common outcome of Prc deficiency in ExPEC strains.

The OMPs of pathogenic bacteria are often involved in the host-bacterium interaction during the course of infections [48]. This fact led us to speculate that the altered OMP expression in the *prc*^−^ ExPEC strains may be responsible for the defect in UT colonization. To determine the OMPs differentially expressed in ExPEC with and without *prc*, the proteomic profiles of WT-RS218 and Δ*prc*-RS218 were determined by liquid chromatography-tandem mass spectrometry (LC/MS/MS). The levels of 25 and 32 proteins were shown to be significantly downregulated and upregulated, respectively, by the *prc* deletion (Additional file 2: Table S2). Among them, 2 OMPs (FliC and SlyB) were upregulated in Δ*prc*-RS218, while 6 OMPs (Spr, BamA, Tsx, TolC, Ag43 and TraT) were downregulated in the *prc* mutant (Table 2).

### Prc deficiency decreases the flagellin level and motility of ExPEC

While identified in the LC/MS/MS analysis, FliC (flagellin) is the major component of the flagellar filament, and flagellum-mediated motility is required for ExPEC to cause UTIs [12–15]. Thus, our study subsequently focused on the FliC protein.

**Fig. 3:**
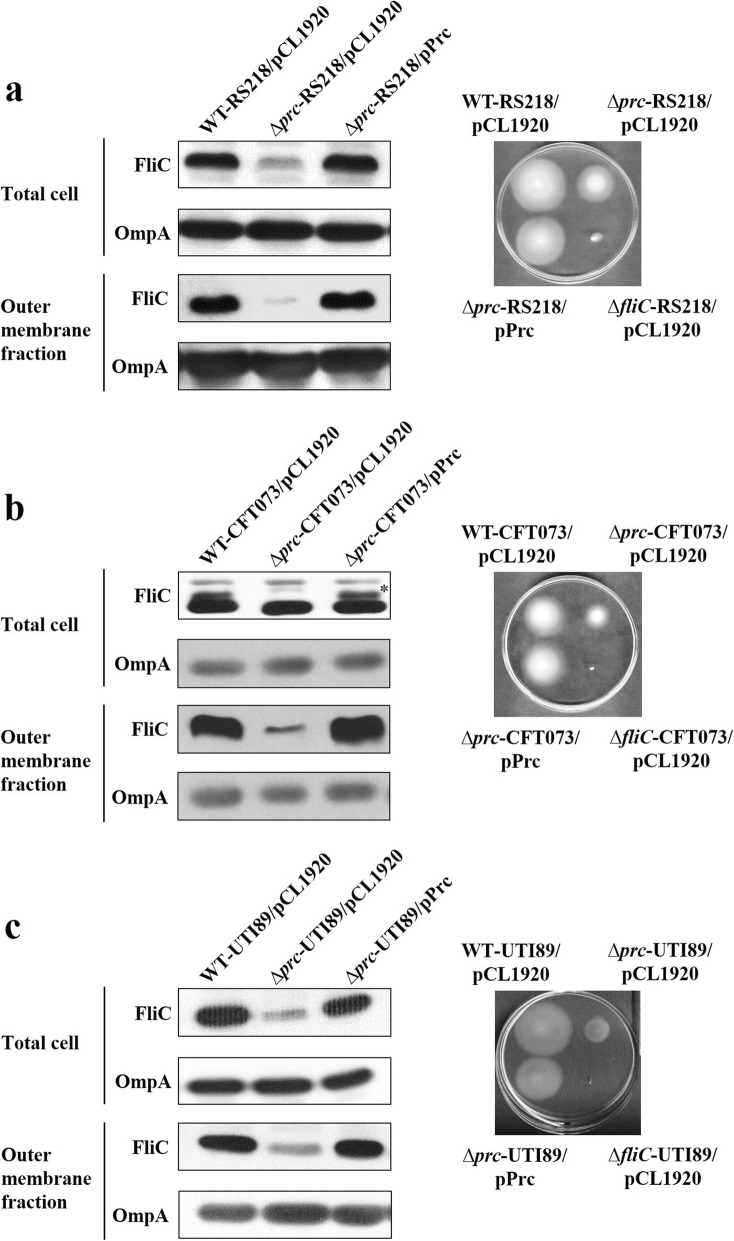
The levels of FliC expression and motility of RS218, CFT073, UTI89, and their *prc* mutants. The effects of *prc* deletion on FliC expression and motility in the ExPEC strains RS218 (**a**), CFT073 (**b**), and UTI89 (**c**). The results of western blot analyses of the total cell lysates and the OM fractions of the indicated bacterial strains are shown in the left panels of (**a**), (**b**), and (**c**). OmpA served as a loading control. The motilities of the indicated strains on 0.3% agar plates are shown in the right panels of (**a**), (**b**), and (**c**). pCL1920, the empty plasmid vector; pPrc, the plasmid pCL1920 harboring *prc* (Table 1). The asterisk indicates the FliC protein in the total lysate of CFT073

To confirm the LC/MS/MS results regarding FliC, the levels of FliC in WT-RS218 and Δ*prc*-RS218 were further determined by western blot analysis with an anti-FliC antiserum. Consistently, Δ*prc*-RS218 showed lower levels of FliC in total bacterial lysate and the OM fraction than WT-RS218 (Fig. 3a left panel). In addition, the *prc* mutant showed a significantly lower motility than the wild-type strain (Fig. 3a right panel). Trans-complementation of Δ*prc*-RS218 with the *prc* gene restored the expression of FliC and motility to the levels of those of WT-RS218. Similar phenotypes were also shown in CFT073 and UTI89 (Fig. 3b and b). These results demonstrate that Prc deficiency reduces flagellin (FliC) expression and suppresses bacterial motility in ExPEC.

### Prc deficiency decreases the expression of the flagellar regulon

**Fig. 4:**
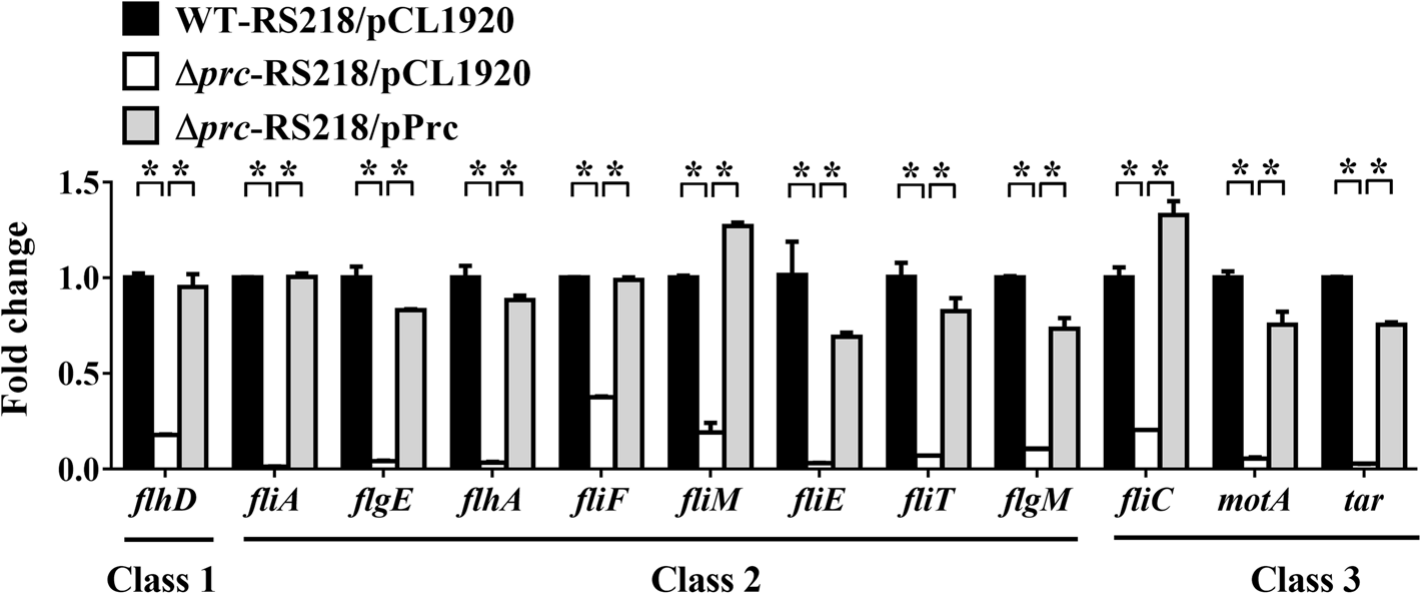
The expression of the flagellar regulon in the wild-type, *prc* mutant, and complemented strains of ExPEC. The transcript levels of the class 1 gene *flhD*, class 2 genes (*fliA, flgE, flhA, fliF, fliM, fliE, fliT* and *flgM*) and class 3 genes (*fliC, motA,* and *tar*) were determined by qPCR. The transcript levels of the genes in each strain, which were normalized to those of the housekeeping gene *ftsZ*, are presented as the relative levels compared to those in WT-RS218/pCL1920. The results were derived from experiments performed in triplicate and are shown as the means ± standard deviations. pCL1920, the empty plasmid vector; pPrc, the plasmid pCL1920 harboring *prc* (Table 1). The asterisks indicate significant differences (*P* values < 0.05) of the comparisons between WT-RS218/pCL1920 and Δ*prc*-RS218/pCL1920 as well as between Δ*prc*-RS218/pCL1920 and Δ*prc*-RS218/pPrc

**Fig. 5:**
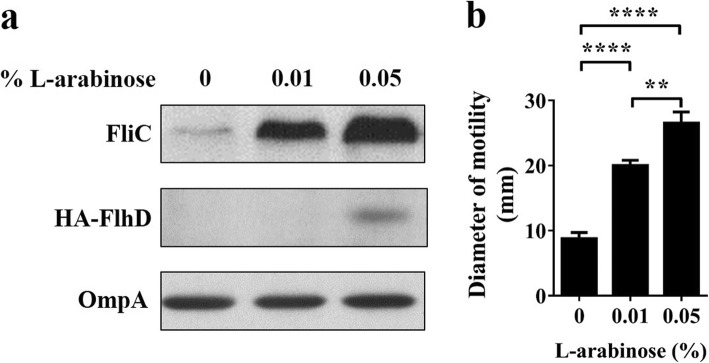
Effects of increased FlhDC expression on the FliC level and motility in the *prc* mutant. FliC and HA-tagged FlhD levels (**a**) and the motility (**b**) of ∆*prc*-RS218/pBAD-FlhDC with different levels of L-arabinose treatment. Each motility quantitative result was derived from experiments performed in triplicate and is presented as the means ± standard deviations. The plasmid pBAD-FlhDC harbored HA-tagged *flhD* and His-tagged *flhC* genes that were under the control of an arabinose-inducible promoter (Table 1). The levels of FliC, FlhD, and OmpA were determined by western blot analyses with a rabbit anti-FliC antiserum and an anti-HA antibody and mouse anti-OmpA antiserum, respectively. The levels of OmpA served as loading controls

*fliC* is located at the lowest level (class 3) of the transcriptional hierarchy of the flagellar regulon [16]. We investigated whether Prc deficiency affects the expression of this regulatory cascade. Δ*prc*-RS218 exhibited lower expression levels of the class 1 (*flhD*), class 2 (*fliE*, *fliF*, *flhA*, *flgE*, *flgM*, *fliM*, *fliT*, and *fliA*) and class 3 (*fliC* and *motA*) genes than WT-RS218 (Fig. 4). Trans-complementation of Δ*prc*-RS218 with *prc* restored the expression of these genes, suggesting that Prc deficiency results in the downregulation of all classes of genes in the regulon. Given that the top master operon *flhDC* governs all genes in this regulon, it is likely that *prc* deletion suppresses *flhDC* expression to cause the reduced motility. To assess this speculation, we examined the motilities of Δ*prc*-RS218 strains with different levels of *flhDC* expression. To do so, ∆*prc*-RS218 was transformed with a *flhDC*-containing plasmid, pBAD-FlhDC (Table 1). Because the *flhDC* operon in this plasmid was driven by an arabinose-inducible promoter, arabinose treatment dose-dependently induced the expression of FlhDC (Fig. 5a). The induction of *flhDC* expression also dose-dependently increased the FliC level and bacterial motility (Fig. 5a and b). These results supported that through suppressing the expression of *flhDC*, *prc* deletion downregulates the whole flagellar regulon and consequently decreases bacterial motility.

### The defective motility is responsible for the decreased ability of the *prc* mutant to cause UTIs

**Fig. 6:**
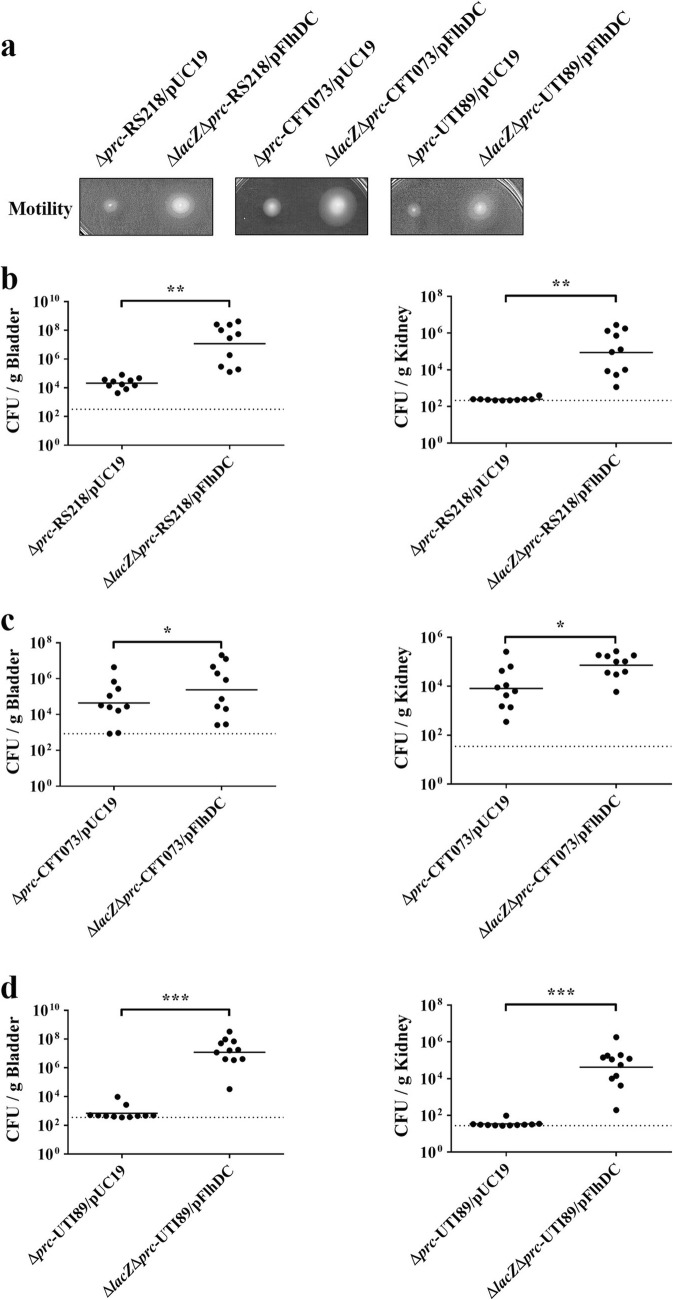
Effect of motility increase on the ability of the ExPEC *prc* mutants to cause UTIs. (**a**) Construction of *prc* mutants constitutively overexpressing *flhDC*. The ∆*lacZ*∆*prc* strains of the ExPEC strains RS218, CFT073, and UTI89 were transformed with the plasmid pFlhDC (Table 1). The *flhDC* operon encoded in this plasmid was fused with a *lac* promoter. Without any induction, the *flhDC* overexpression driven by leaky promoter activity was strong enough to improve the bacterial motility of the *prc* mutants. (**b**, **c**, and **d**) Transurethral cochallenge of mice with the *prc* mutants of the indicated ExPEC strains with and without *flhDC* overexpression. At 48 hpi, the bacterial counts in the bladders and kidneys were enumerated and differentiated by spreading the homogenized infected tissue onto LB agar plates with IPTG and X-gal. Horizontal bars indicate the median level of the bacterial counts. The dotted line represents the limit of detection

To further investigate whether the defective motility caused by *prc* deletion contributes to the attenuated ability to cause UTIs, we promoted the motility of *prc*^−^ ExPEC strains and then assessed their abilities to cause UTIs. The plasmid pFlhDC, which can constitutively overexpress *flhDC*, was introduced into the *prc*^−^ strains of RS218, CFT073, and UTI89 and was able to increase their motilities (Fig. 6a). The pFlhDC-harboring *prc* mutants showed higher levels colonization than the corresponding empty vector-harboring strains (Fig. 6b, c, and d). These results demonstrated that the decreased motility is responsible for the defective ability of *prc* mutants to colonize the UT.

### Deficiency in Prc increases the expression of the genes governed by some ESSSs

**Fig. 7:**
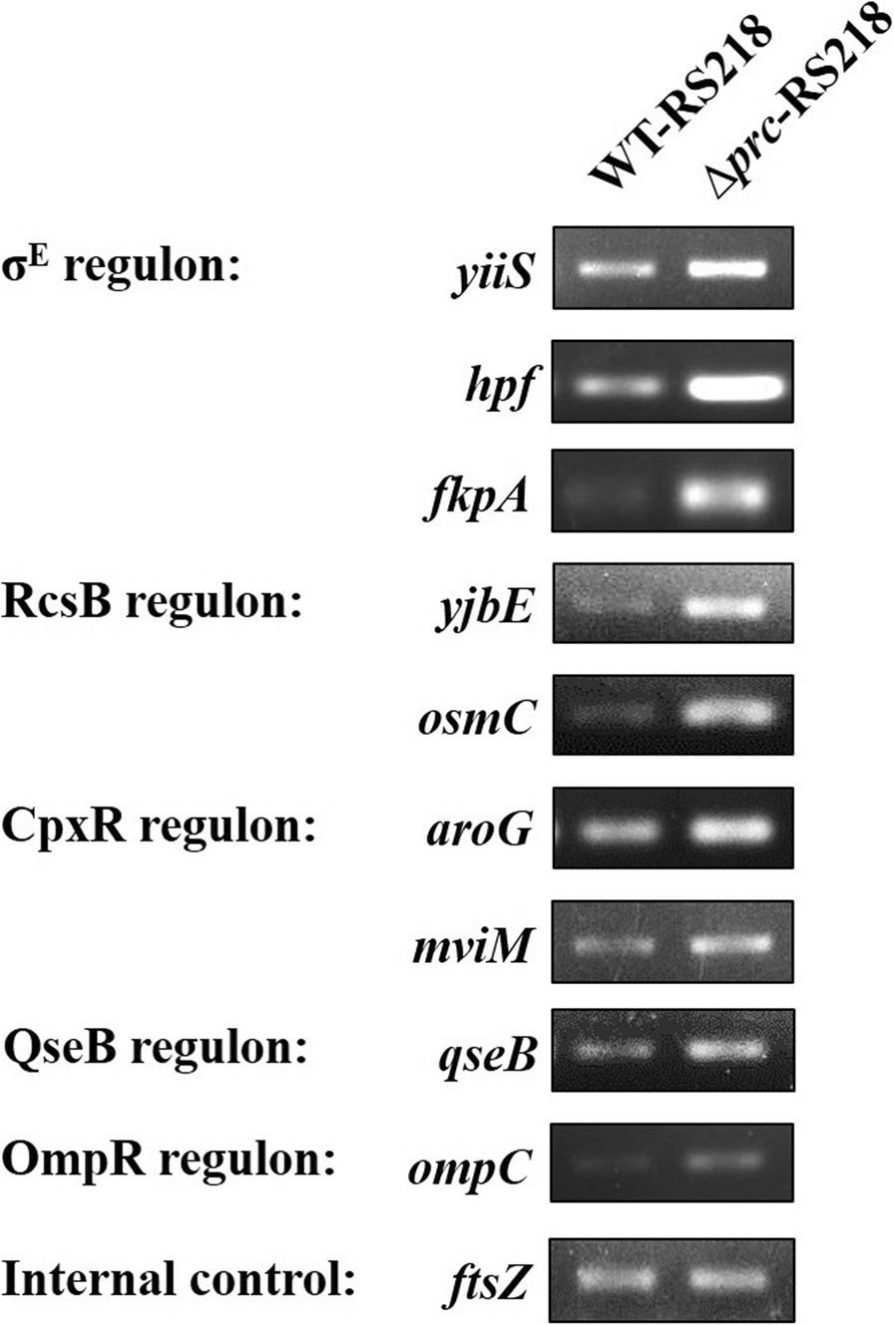
The expression of the genes positively regulated by the extracytoplasmic signaling systems in WT-RS218 and Δ*prc*-RS218. The transcriptional levels of the genes in the σ^E^ (*yiiS*, *hpf*, and *fkpA*), RcsCDB (*yjbE* and *osmC*), CpxA-CpxR (*aroG* and *mviM*), QseB-QseC (*qseB*), and EnvZ-OmpR (*ompC*) regulons [22, 49–55] were determined by RT-PCR and visualized on agarose gels after EtBr staining. *ftsZ* served as a housekeeping gene internal control

To investigate how Prc deficiency results in the downregulation of *flhDC* transcription, comparative transcriptome analysis of ∆*prc*-RS218 and WT-RS218 by RNA-seq was performed. The *prc* deletion significantly affected the transcriptome of ExPEC, in which 152 and 365 genes were upregulated and downregulated, respectively (Additional file 3: Table S3). Consistently, many genes in the flagellar regulon were shown to be significantly downregulated in the *prc* mutant (Table 3). In addition, genes known to be able to be upregulated by some extracytoplasmic stress signaling systems (ESSSs), including the σ^E^, RcsCDB, CpxA-CpxR, QseB-QseC, and EnvZ-OmpR systems, showed significantly higher expression levels in ∆*prc*-RS218 than in WT-RS218 (Table 3). The differential transcript levels of the representative genes governed by these ESSSs were further confirmed by RT-PCR analysis (Fig. 7). These findings suggest that these ESSSs may be activated in the *prc* mutant. It is likely that the activated ESSSs contribute to the reduced motility.

### Blocking the activation of the σ^E^ or RcsCDB system partially restores the motility of the *prc* mutant of ExPEC

**Fig. 8:**
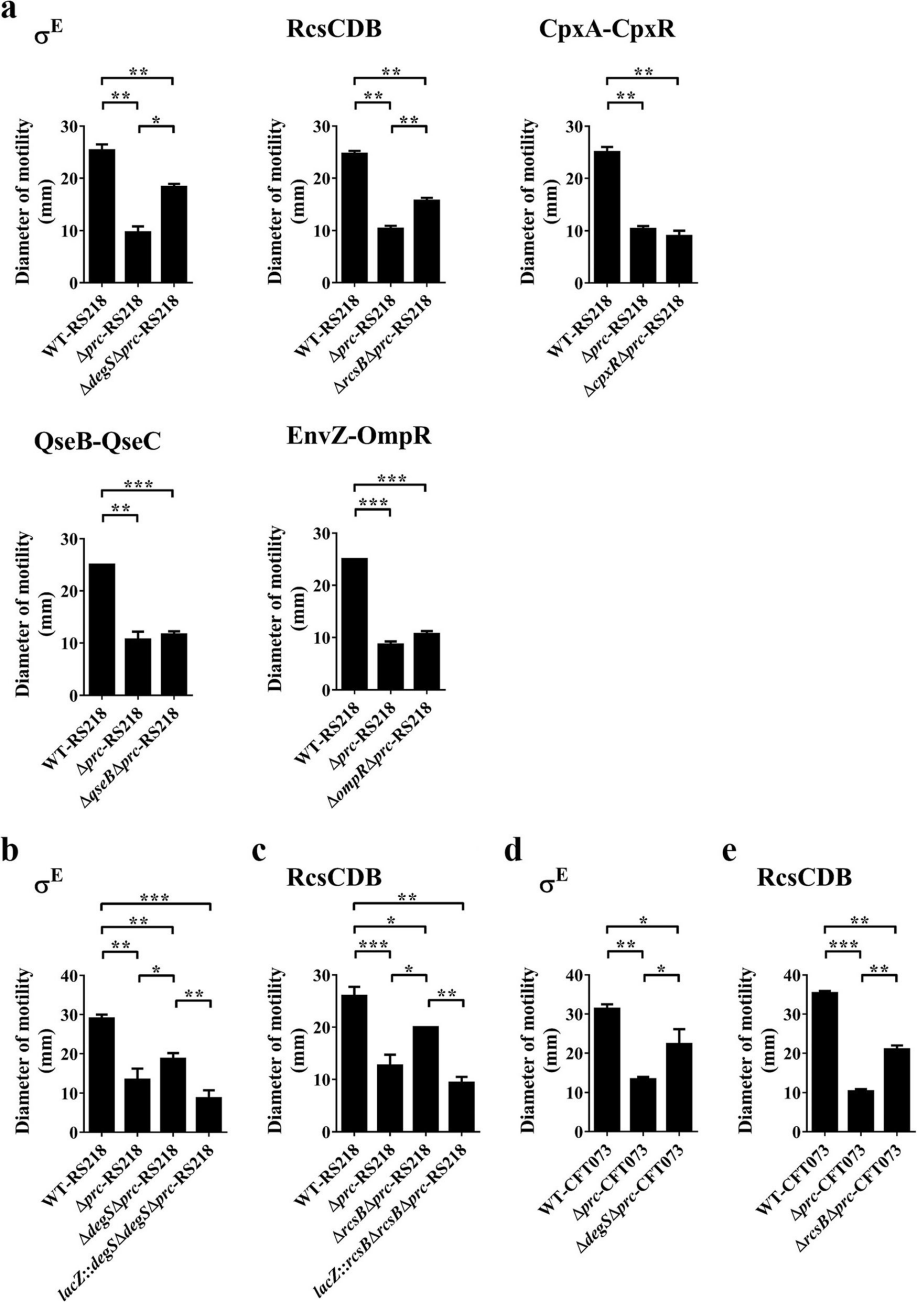
The motilities of the *prc* mutant strains with inactivated extracytoplasmic signaling pathways. (**a**) The motilities of the *prc* mutant strains of RS218 with a blocked σ^E^, RcsCDB, CpxA-CpxR, QseB-QseC, or EnvZ-OmpR system. *degS*, *rcsB*, *cpxR*, *qseB*, and *ompR* deletions were introduced into Δ*prc*-RS218 to block the σ^E^, RcsCDB, CpxA-CpxR, QseB-QseC, and EnvZ-OmpR signaling pathways, respectively. (**b**) The motility of the Δ*degs*Δ*prc*-RS218 strain complemented with *degS* in the chromosomal *lacZ* locus. (**c**) The motility of the Δ*rcsB*Δ*prc*-RS218 strain complemented with *rcsB* in the chromosomal *lacZ* locus. (**d** and **e**) The motilities of the *prc* mutant strains of CFT073 with an inactivated σ^E^ (**d**) or RcsCDB (**e**) system

If the ESSSs play roles in suppressing the motility of the *prc* mutant, blocking their activation may relieve the suppression. As deletion of the *degS*, *rcsB*, *cpxR*, *qseB*, and *ompR* genes blocks the activation of the σ^E^, RcsCDB, CpxA-CpxR, QseB-QseC, and EnvZ-OmpR systems [35, 49, 50, 56, 57], respectively, the deletion of these genes was introduced into Δ*prc*-RS218 to inactivate the corresponding systems. As shown in Fig. 8a, the *degS* and *rcsB* deletions partially restored the motility of Δ*prc*-RS218, while the *cpxR*, *qseB*, and *ompR* deletions showed no significant effect on bacterial motility. Complementation of the double mutant strains Δ*degs*Δ*prc*-RS218 and Δ*rcsB*Δ*prc*-RS218 with *degS* and *rcsB* in the chromosomal *lacZ* locus, respectively, decreased the motilities of these strains to the level of that of the *prc* single mutant (Fig. 8b and c), suggesting that activation of the σ^E^ and RcsCDB systems is involved in the decreased motility of the *prc* mutant. Similar results were also shown in CFT073 (Fig. 8d and e).

### The activated σ^E^ and RcsCDB systems are involved in the suppression of FliC expression and *flhDC* transcription in Δ*prc*-RS218

**Fig. 9:**
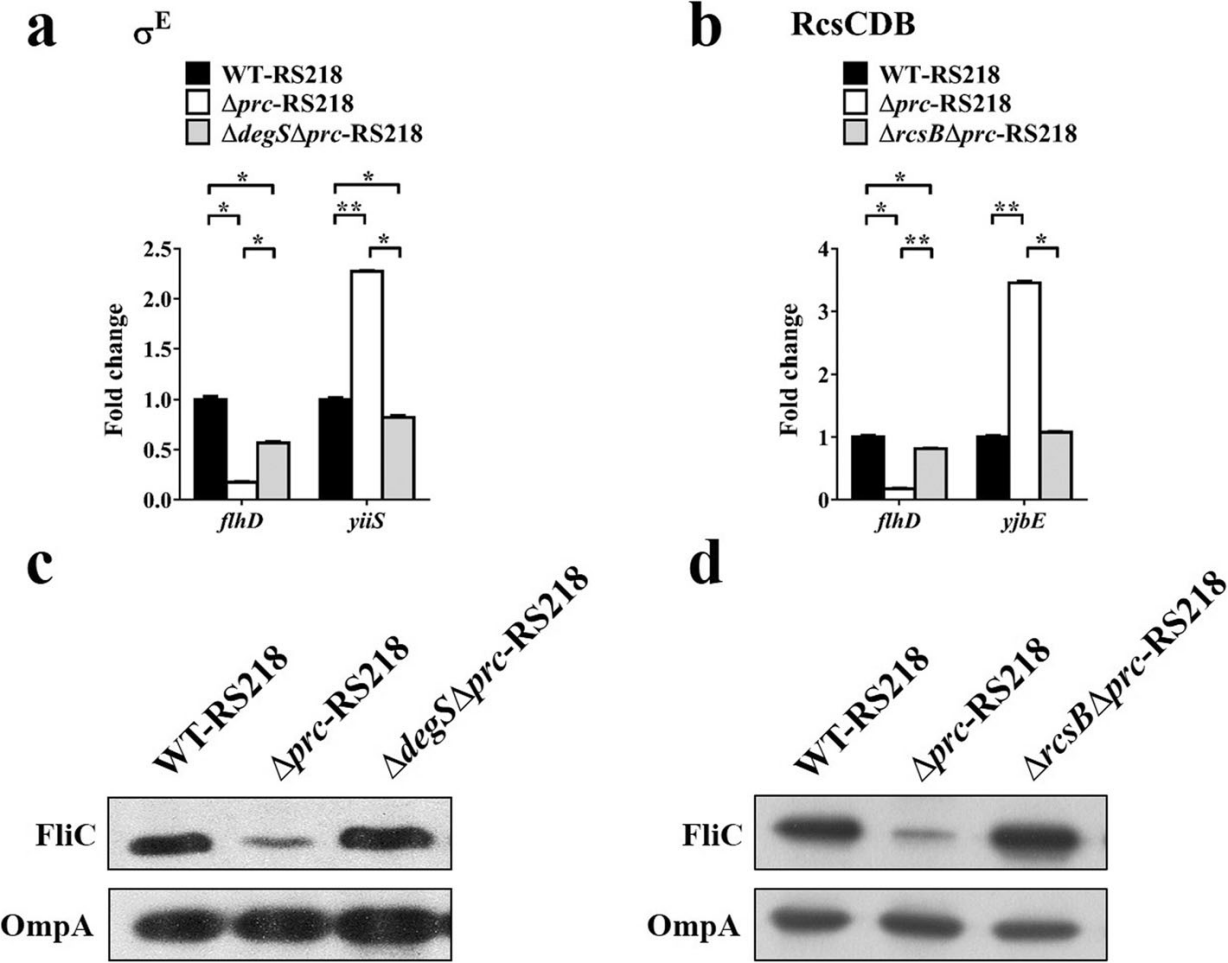
The effects of σ^E^ and RcsCDB inactivation on the transcript levels of the *flhDC* operon and the FliC levels in the *prc* mutant. (**a** and **b**) The mRNA levels of *flhD, yiiS*, and *yjbE* in the indicated strains determined by qPCR. The levels of *yiiS* and *yjbE* reflect the activation levels of the σ^E^ and RcsCDB systems, respectively. The mRNA level of each gene in a strain was normalized to the *ftsZ* level and presented as a relative level compared to that in WT-RS218. The results were derived from experiments performed in triplicate and are shown as the means ± standard deviations. (**c** and **d**) The levels of FliC in the indicated strains were determined by western blot analysis with a rabbit anti-FliC antiserum. OmpA levels served as loading controls

We further investigated whether inactivation of the σ^E^ or RcsCDB systems could restore the expression of *flhDC* and FliC in the *prc* mutant. *yiiS* and *yjbE* are the genes positively regulated by the σ^E^ and RcsCDB systems, respectively. Thus, the expression levels of these genes can reflect the activation levels of the corresponding signaling systems [51, 52]. In comparison with Δ*prc*-RS218, the double mutants Δ*degs*Δ*prc*-RS218 and Δ*rcsB*Δ*prc*-RS218 showed lower expression of *yiiS* and *yjbE* (Fig. 9a and b). This result indicates that the *prc* deletion-induced σ^E^ and RcsCDB activation was blocked in Δ*degs*Δ*prc*-RS218 and Δ*rcsB*Δ*prc*-RS218, respectively. In addition, in comparison with Δ*prc*-RS218, both Δ*degs*Δ*prc*-RS218 and Δ*rcsB*Δ*prc*-RS218 showed increased levels of *flhDC* (Fig. 9a and b) and FliC expression (Fig. 9c and d). These results suggest that the activated σ^E^ or RcsCDB systems contribute to the suppressed flagellar expression, resulting in the defective motility in the *prc* mutant.

### The activated σ^E^ system suppresses motility and *flhDC* transcription in wild-type ExPEC

**Fig. 10:**
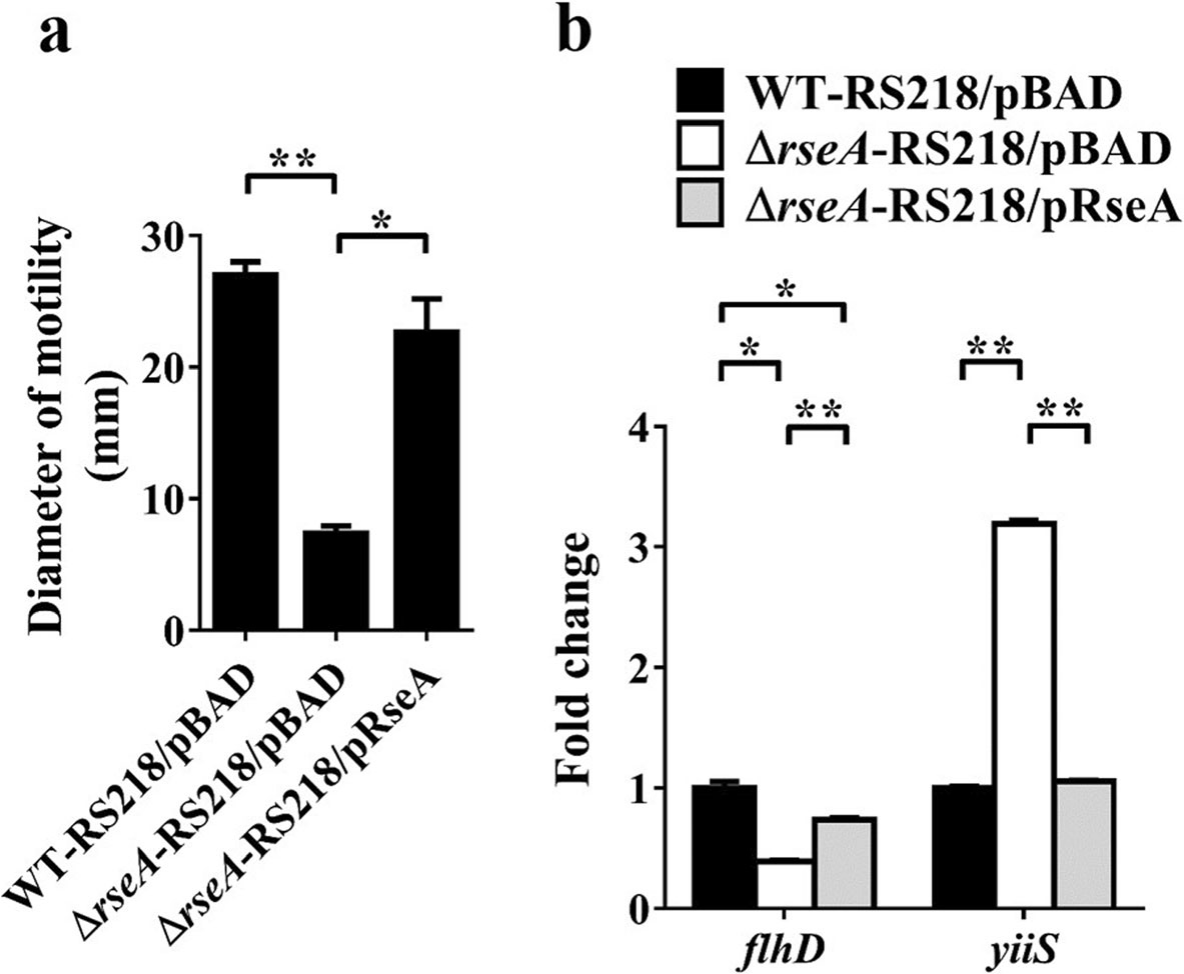
The effect of σ^E^ activation on the motility and transcript level of *flhD* in the *prc*^*+*^ ExPEC strain. (**a**) Motility diameters of the indicated strains. (**b**) Relative mRNA levels of *flhD* and *yiiS* determined by qPCR. The *yiiS* gene served as the reporter of σ^E^ activation. The mRNA level of each gene in a strain was normalized to the *ftsZ* level and presented as a relative level compared to that in WT-RS218 harboring pBAD (WT-RS218/pBAD). Arabinose (0.2%) was used to induce the expression of RseA that was encoded in the pRseA plasmid (Table 1) and driven by the arabinose-inducible promoter on the plasmid. The results were derived from experiments performed in triplicate and are shown as the means ± standard deviations. pRseA, pBAD harboring *rseA*

It has been previously shown that activation of the RcsCDB system can downregulate the expression of *flhDC* and thus suppress motility in *E. coli* without *prc* mutation [21, 24]. This finding suggests that the activation of the RcsCDB signaling pathway alone in the *prc* mutant strain is sufficient to cause the suppression, requiring no other signals induced by the *prc* mutation. To the best of our knowledge, this report is the first study demonstrating that the σ^E^ system is involved in motility suppression in the *prc* mutant of *E. coli* (Fig. 8a, b, and d). However, it remained unclear whether activation of the σ^E^ system could suppress bacterial motility in an *E. coli* strain with an intact Prc. We assessed the motility and *flhDC* expression of a *prc*^+^ RS218 strain (Δ*rseA*-RS218) in which the σ^E^ system was activated by deleting the anti-σ^E^ factor RseA [58]. As shown in Fig. 10a and b, activation of the σ^E^ system in the *prc*^+^ background significantly decreased bacterial motility and suppressed *flhDC* expression. These findings suggested that without other *prc* deletion-induced signals, activation of the σ^E^ system was sufficient to suppress *flhDC* expression and thus bacterial motility in the *prc* mutant.

### The σ^E^ and RcsCDB systems can work independently to downregulate bacterial motility

**Fig. 11:**
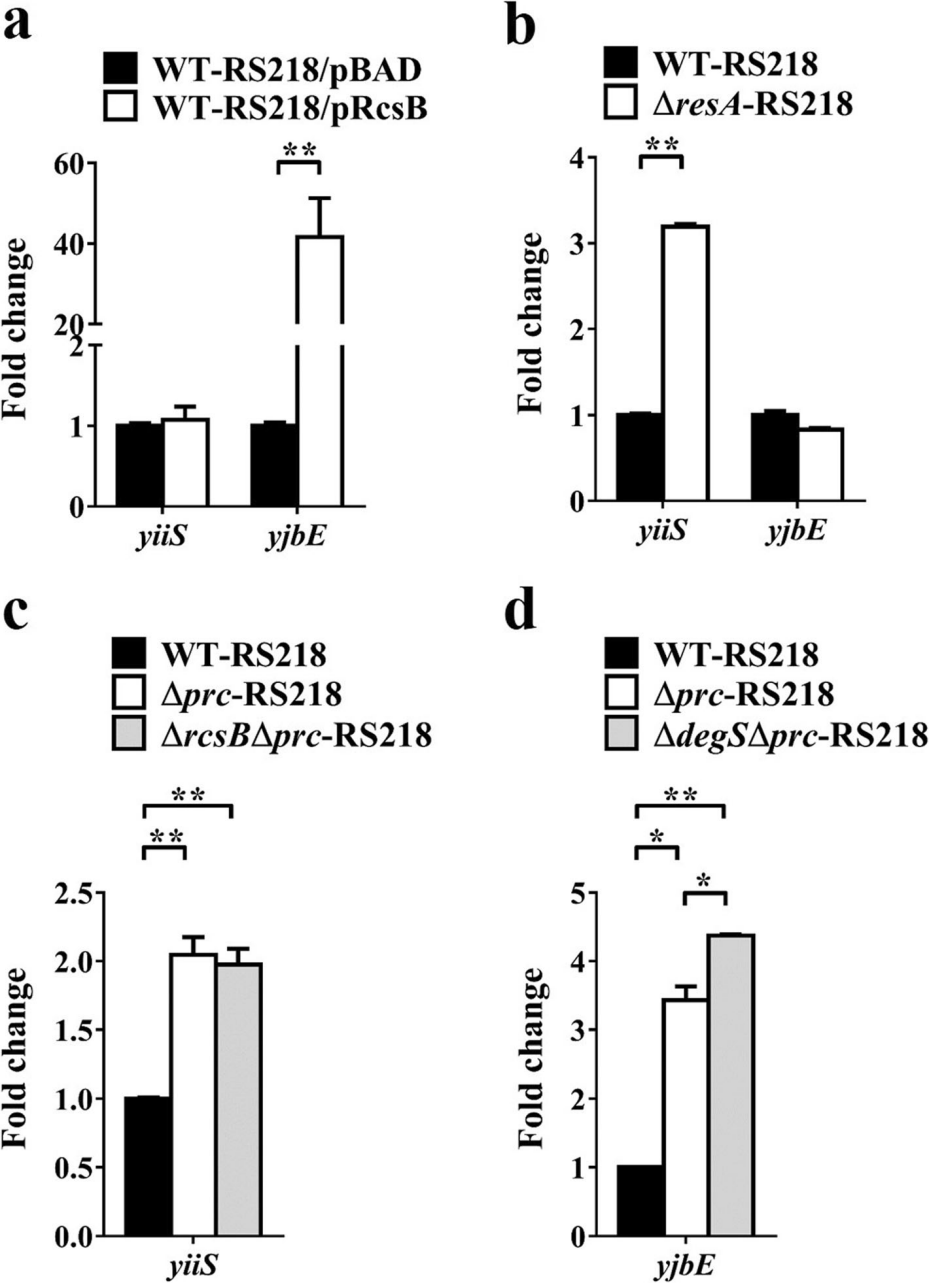
The activated σ^E^ and RcsCDB systems can work independently to suppress bacterial motility. (**a**) The mRNA levels of the σ^E^-regulated gene *yiiS* and the RcsCDB-regulated gene *yjbE* in the RS218 strain with an unactivated or activated RcsCDB system. Arabinose (0.2%) was used to induce the overexpression of RcsB encoded in pRcsB (Table 1). (**b**) The mRNA levels of *yiiS* and *yjbE* in the bacteria with an unactivated or activated σ^E^ system. (**c**) The mRNA levels of *yiiS* in WT-RS218, Δ*prc*-RS218, and Δ*rcsB*Δ*prc*-RS218. (**d**) The mRNA levels of *yjbE* in WT-RS218, Δ*prc*-RS218, and Δ*degS*Δ*prc*-RS218. The mRNA level of each gene, which was determined by qPCR, in a strain was normalized to the *ftsZ* level and presented as a relative level compared to that in WT-RS218. The results were derived from experiments performed in triplicate and are shown as the means ± standard deviations. pRcsB, pBAD harboring the *rcsB* gene driven by the arabinose-inducible promoter on the plasmid

We investigated whether the activation of the σ^E^ and RcsCDB systems in ExPEC are sequential (upstream and downstream) events. It has been shown that RcsB overexpression triggers the activation of the RcsCDB system [59]. RS218 was transformed with the RcsB-overexpressing plasmid pRcsB (WT-RS218/pRcsB) to create a RcsCDB-activated strain. WT-RS218/pRcsB showed significantly higher *yjbE* expression than RS218 harboring an empty plasmid vector (WT-RS218/pBAD), while the two strains showed similar levels of *yiiS* expression (Fig. 11a). This result suggested that activating the RcsCDB system does not trigger the activation of the σ^E^ system. On the other hand, the σ^E^-activated strain Δ*rseA*-RS218 showed significantly higher *yiiS* expression than WT-RS218, while these two strains showed similar levels of *yjbE* expression (Fig. 11b). This finding suggested that activation of the σ^E^ system does not trigger the activation of the RcsCDB system. Collectively, these results suggested that the activation of these systems is independent of each other in ExPEC.

We further determined whether the two systems are also independently activated by Prc deficiency. Δ*rcsB*Δ*prc*-RS218, in which the activation of the RcsCDB system was blocked, and Δ*prc*-RS218 showed a similar level of *yiiS* expression (Fig. 11c), suggesting that the RcsCDB signal does not contribute to the activation of the σ^E^ system in the *prc* mutant. The σ^E^-inactivated strain Δ*degS*Δ*prc*-RS218 showed a slightly higher level of *yjbE* expression than Δ*prc*-RS218 (Fig. 11d), suggesting that σ^E^ does not contribute to RcsCDB system activation in the *prc* mutant. Taken together, these results indicate that Prc deficiency independently induces the activation of the σ^E^ and RcsCDB systems.

### The protease activity of Prc is required for bacterial motility and FliC expression

**Fig. 12:**
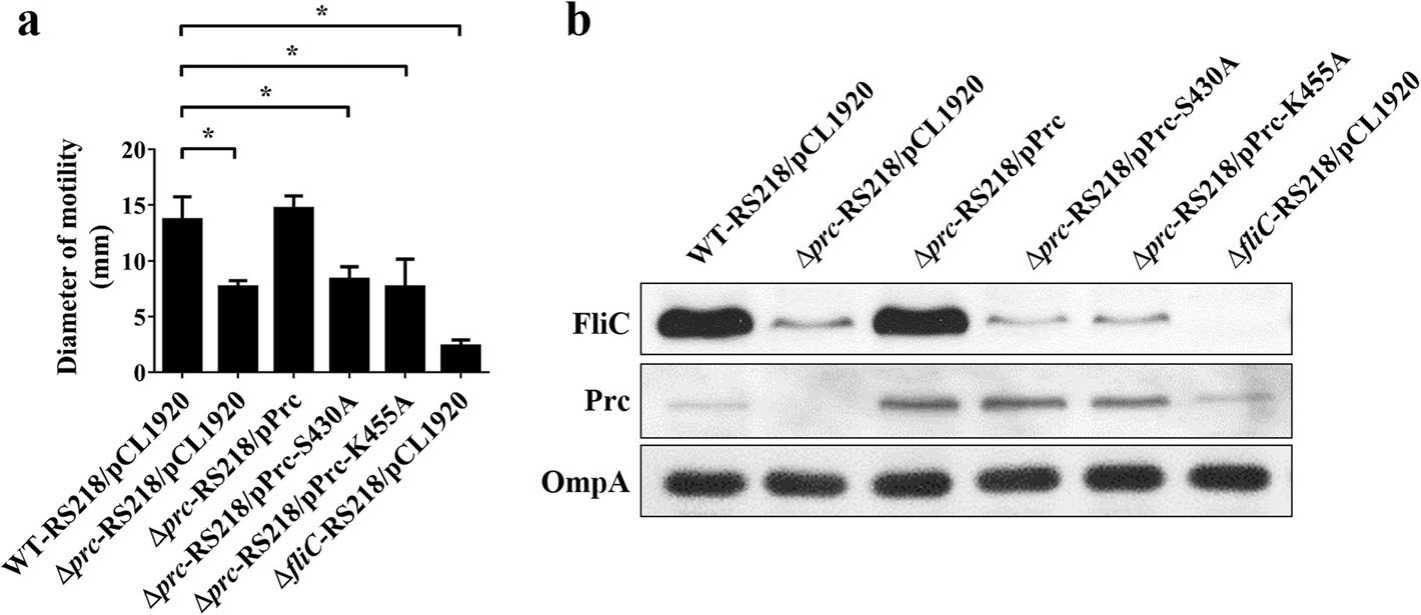
A lack of Prc protease activity was responsible for the defective motility and decreased FliC expression in the *prc* mutant. (**a**) Motility diameters of the strains expressing wild-type Prc, Prc S430A, and Prc K455A. The results were derived from three independent experiments and are shown as the means ± standard deviations. (**b**) The protein levels of FliC, Prc, and OmpA in the bacteria expressing wild-type Prc, Prc S430A, and Prc K455A. The protein levels were determined by western blot analysis with a rabbit anti-FliC antiserum, rabbit anti-Prc antiserum and mouse anti-OmpA antiserum, respectively. The OmpA level served as a loading control. Δ*fliC*/pCL1920 served as a nonmobile and non-FliC expression control. pCL1920, the plasmid pCL1920, which served as an empty vector control. pPrc, the plasmid pCL1920 harboring *prc*; pPrc-S430A, the plasmid pCL1920 harboring a mutated *prc* expressing Prc S430A; pPrc-K455A, the plasmid pCL1920 harboring a mutated *prc* expressing Prc K455A

Since Prc is a protease, we investigated whether the deficiency of the Prc protease function contributes to the decreased motility and FliC expression in the *prc* mutant. Prc variants with a S430A or K455A substitution are known to lose catalytic activity but still maintain the original protein structure and substrate-binding ability [60]. Trans-complementation of Δ*prc*-RS218 with the catalytic ability-defective variants failed to restore the motility and FliC level (Fig. 12a and b). These results suggested that deficiency in the Prc protease activity is responsible for the reduced flagellum expression and thus the defective motility.

### The accumulation of Spr is responsible for the defective motility and decreased FliC expression in Δ*prc*-RS218

**Fig. 13:**
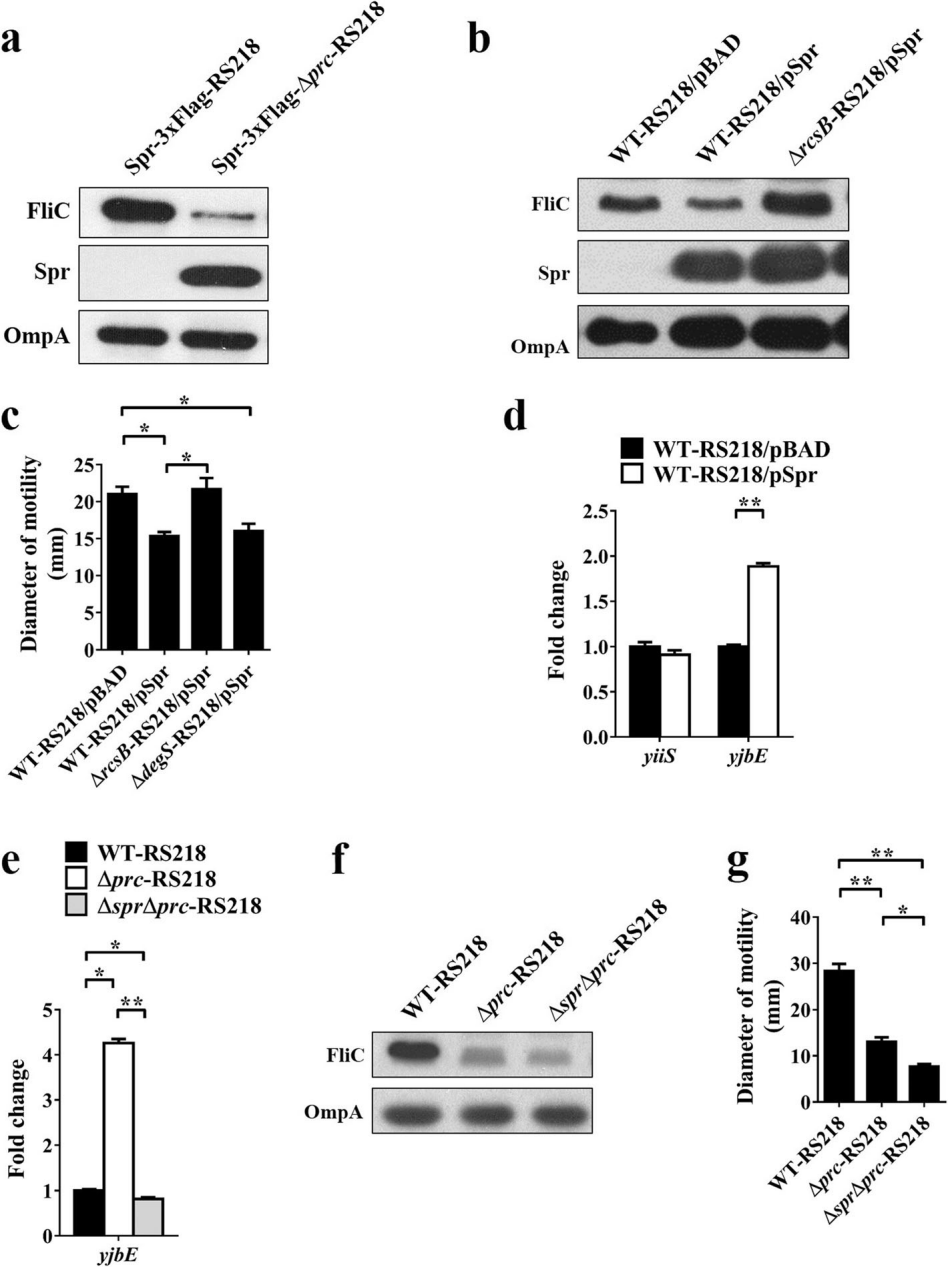
Effects of Spr accumulation on bacterial motility, FliC expression and the activation of the extracytoplasmic signaling systems. (**a**) The levels of Spr and FliC in the RS218 strains with or without *prc*. To detect Spr, these strains were modified to express C-terminally 3xFlag-tagged Spr. Western blot analyses with an anti-Flag antibody and a rabbit anti-FliC antiserum were performed to detect the protein levels. The OmpA levels served as loading controls, which were probed with a mouse anti-OmpA antiserum. Spr-3xFlag-RS218, the RS218 strain with the native chromosomal *spr* fused with a sequence encoding a 3xFLAG tag at the 3′ end; Spr-3xFlag-Δ*prc*-RS218, the Δ*prc*-RS218 strain with the chromosomal *spr* fused with a sequence encoding a 3xFLAG tag at the 3′ end. (**b**) The FliC and Spr levels in the RS218 strains with or without the overexpression of the recombinant Spr, which was C-terminally fused with a Flag tag. The Spr protein was detected with an anti-Flag antibody. Arabinose (0.2%) was used to trigger the expression of the recombinant Spr that was encoded in pBAD and driven by the arabinose-inducible promoter in the plasmid. pSpr, pBAD harboring *spr* fused with a sequence encoding a Flag tag at the 3′ end. (**c**) Motility diameters of the strains overexpressing Spr. (**d**) The relative mRNA levels of *yiiS* and *yjbE* compared to those in WT-RS218/pBAD. (**e**) The relative *yibE* levels compared to those in WT-RS218. (**f**) The FliC level in WT-RS218, Δ*prc*-RS218, and Δs*pr*Δ*prc*-RS218. (**g**) Motility diameters of WT-RS218, Δ*prc*-RS218, and Δs*pr*Δ*prc*-RS218

Given that a protease exerts biological function through mainly proteolytic regulation of its substrates, we reasoned that the dysregulation of Prc substrates due to the deficiency in the protease activity may be responsible for the reduced motility in the *prc* mutant. The Prc substrate Spr has been shown to accumulate in a *prc* mutant of the commensal *E. coli* MG1655 [38]. Similarly, our proteome data showed that Δ*prc*-RS218 expressed a significantly higher level of Spr than WT-RS218 (Table 2). A western blot analysis of Spr in the RS218 strains with or without *prc* further confirmed this finding (Fig. 13a), suggesting that Spr accumulation is common among the *E. coli* strains in which Prc is inactivated. We overexpressed Spr to raise the intracellular level of Spr in RS218. Spr overexpression significantly decreased the FliC level (Fig. 13b) and bacterial motility (Fig. 13c), suggesting that Spr accumulation in the *prc* mutant contributes to reduced motility.

To determine whether Spr accumulation in ExPEC triggers activation of the RcsCDB and σ^E^ systems, the transcription levels of the RcsCDB-regulated *yjbE* and σ^E^-regulated *yiiS* genes in an Spr-overexpressing strain (WT-RS218/pSpr) and a strain with normal Spr expression (WT-RS218/pBAD) were determined (Fig. 13d). Overexpression of Spr upregulated *yjbE*, but *yiiS* was not affected. This suggested that Spr accumulation triggers activation of the RcsCDB system but not the σ^E^ system. Consistent with this finding, blocking the activation of RcsCDB signaling by deleting *rcsB* significantly increased the motility of the Spr-overexpressing strain, while blocking σ^E^ signaling by deleting *degS* did not affect bacterial motility (Fig. 13c). Additionally, deletion of *rcsB* increased the FliC level in the Spr-overexpressing strain (Fig. 13b). These results suggest that Spr accumulation in the *prc* mutant triggers activation of the RcsCDB system and thus suppresses flagellin expression and bacterial motility.

In addition, we further investigated whether blocking Spr accumulation in the *prc* mutant affects the activation of the RcsCDB system and motility. We blocked Spr accumulation in the *prc* mutant by deleting the *spr* gene (Δ*spr*Δ*prc*-RS218). Similar to wild-type RS218, Δ*spr*Δ*prc*-RS218 showed significantly lower *yibE* expression than Δ*prc*-RS218 (Fig. 13e), further supporting that Spr accumulation results in the activation of the RcsCDB system in the *prc* mutant. However, the FliC level (Fig. 13f) and motility (Fig. 13g) of Δ*spr*Δ*prc*-RS218 were not significantly higher than those of Δ*prc*-RS218. These results suggest that deleting *spr* in the *prc* mutant may cause other pleotropic effects that are able to downregulate FliC expression and motility, which can offset the effects of the downregulated RcsCDB activation.

## Discussion

**Fig. 14:**
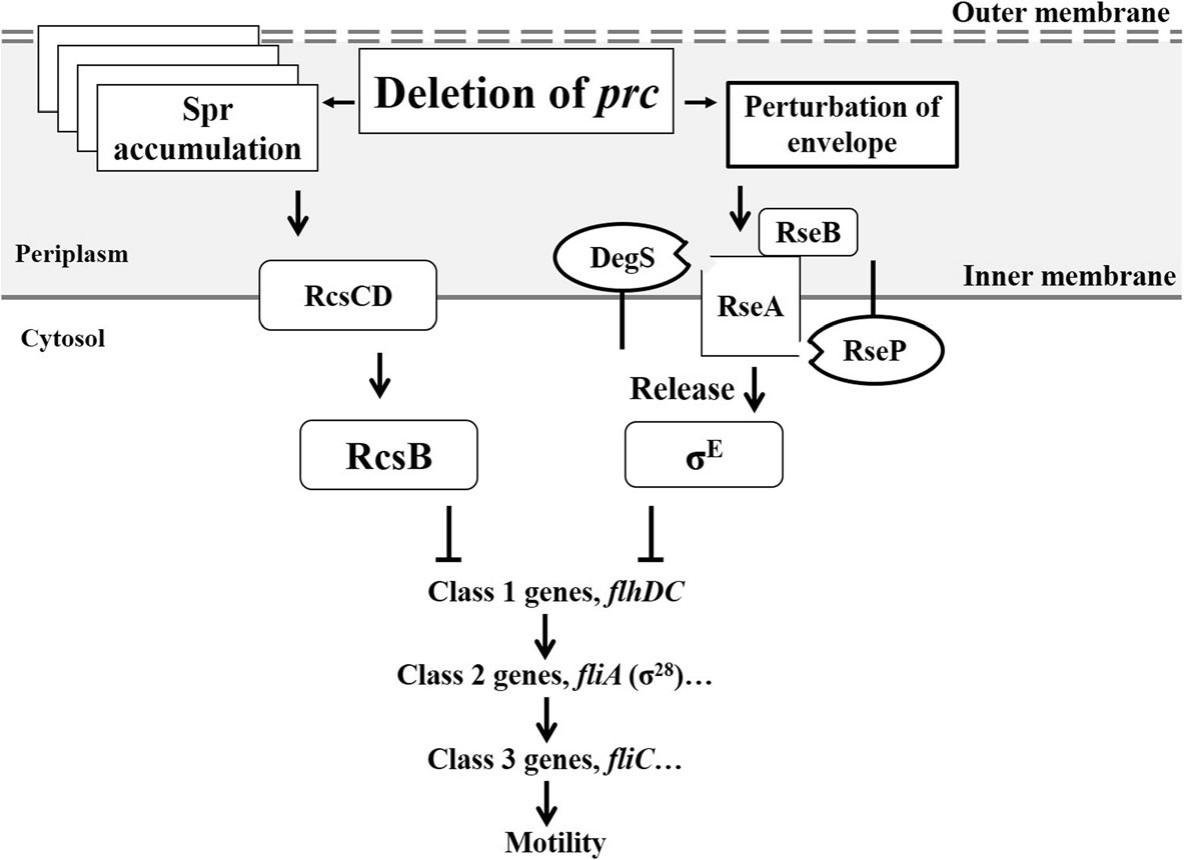
The model describing how deficiency in the Prc protease leads to defective bacterial motility in *E. coli*. Prc protease deficiency interferes with proteolytic regulation of its substrates in the bacterial envelope, leading to uncontrolled levels of Prc substrates, such as Spr. The accumulation of the substrates may alter the OMP profile, leading to compromised OM integrity and disturbing peptidoglycan biogenesis. The resulting disturbance in the envelope triggers activation of the σ^E^ or RcsCDB extracytoplasmic stress response systems. The activated σ^E^ and RcsCDB systems decrease flagellar biosynthesis and thus bacterial motility

This study reveals for the first time that the periplasmic protease Prc in ExPEC contributes to the pathogenesis of UTIs through maintaining intact bacterial motility, which is required for ExPEC to colonize the bladder and kidney [12–15]. In addition, the mechanism of how Prc deficiency interferes with the motility in ExPEC has been elucidated (see the model in Fig. 14). Lack of Prc in the bacteria triggers σ^E^ and RcsCDB signaling, which in turn negatively regulate the expression of the master operon *flhDC* of the flagellar regulon, leading to decreased flagellum expression and hindered bacterial motility. To our knowledge, this report is also the first study demonstrating that σ^E^ signaling negatively regulates the expression of flagella, while activated RcsCDB signaling has previously been shown to be able to suppress this bacterial structure [21]. The accumulation of Spr, a Prc substrate, is responsible for the activated RcsCDB signaling in the ExPEC *prc* mutant. These results demonstrate that Prc-mediated proteolytic regulation of the intracellular substrate is critical for sufficient ExPEC motility to cause UTIs.

The RcsCDB system monitors damage of the OM and peptidoglycan layer [24, 61–64]. It is highly likely that the uncontrolled Spr level in the *prc* mutant activates the RcsBCD system through altering the peptidoglycan structure and interfering with OM integrity. This notion is consistent with the known physical function of Spr and the phenotype resulting from the overexpression of Spr in *E. coli* [65, 66]. Spr is an OM protein [67] and a peptidoglycan hydrolase involved in maintaining the stability of the bacterial peptidoglycan structure [65]. It has been shown that overexpression of Spr in *E. coli* interferes with peptidoglycan biogenesis, leading to decreased peptidoglycan crosslinkage with the OM, which consequently destabilizes the bacterial envelope [66]. In addition, the high level of Spr located on the OM may be a contributing factor to the compromised OM integrity in the *prc* mutant of ExPEC.

Dual molecular signals are required to activate the σ^E^ signaling system: the periplasmic accumulation of denatured OMPs and LPS [36, 68]. Thus, the activation of this signaling system in the *prc* mutant suggests that Prc deficiency causes the accumulation of denatured OMPs and LPS in the periplasmic space. In agreement with this hypothesis, we found that overexpression of DegQ, which is a periplasmic chaperone able to renature misfolded proteins in the periplasm [69], could downregulate the σ^E^ activation and upregulate the FliC expression and motility of the *E. coli prc* mutant (Additional file 4: Figure S1). In addition, construction of the OM requires the transport of OMPs and LPS through the periplasmic space into the OM. In the *prc* mutant, the periplasmic accumulation of these components suggests that Prc deficiency may hinder their transport from the periplasm to the OM. The global proteomic analysis showed that the *prc* mutant (Δ*prc*-RS218) exhibited a significantly higher level of BamA expression than the wild-type strain (Table 2). BamA is the essential component of the β-barrel protein assembly machinery (BAM complex), which is responsible for the assembly of OMPs and the LPS transporter proteins in the OM [70, 71]. The increased BamA expression may be a compensatory response for the mutant since the complex is required for the transport of the OMPs and LPS transporters from the periplasm to the OM.

In the *prc* mutant, both the RcsCDB and σ^E^ systems suppressed bacterial motility through downregulating the expression of *flhDC*. It has been known that RcsB can directly bind to the promoter of *flhDC* to suppress its transcription when the RcsCDB system is activated [21]. However, how σ^E^ signaling downregulates this operon remains to be elucidated. The RNA polymerase holoenzyme (holo-RNAP) is composed of an σ subunit and a core RNA polymerase (co-RNAP), which are responsible for promoter selectivity and RNA synthesis, respectively. In bacterial cells, different types of σ factors compete for a limited pool of common co-RNAP to transcribe a set of genes driven by their cognate promoters [72]. Transcription of the *flhDC* operon is dependent on the housekeeping sigma factor σ^70^ [73]. It is likely that in the *prc* mutant, the activated σ^E^ system allows the σ^E^ factor to compete with σ^70^ for co-RNAP, thus resulting in the downregulation of the *flhDC* operon. Alternatively, σ^E^ may trigger the expression of unknown bacterial factors capable of downregulating *flhDC* transcription.

The present study demonstrates that constitutive activation of σ^E^ and RcsCDB signaling attenuates ExPEC in UTIs through suppressing flagellum-mediated motility. However, it has been shown that blocking σ^E^ signaling will also reduce the bacterium’s ability to cause UTIs [74]. These findings suggest that a tunable σ^E^ signaling system rather than a constantly activated or silenced one is required for the whole virulence of the pathogen during infections. To cause UTIs, ExPEC need to accomplish multiple pathogenic steps, such as adhering to the epithelium lining of the UT, disseminating within the UT, and evading the host immune responses, etc. [75]. The existence of a certain bacterial factor may benefit one pathogenic step but hinder another. Strict regulation of the expression of such factors may also be required for the whole virulence of the pathogen. While flagella are necessary for *E. coli* to disseminate within the UT during infections, flagellin, the major component of flagella, is a potent immunogen able to activate the immune response via TLR5, which may cause the clearance of the invading pathogens. Therefore, σ^E^ signaling may need to remain tunable so that it can work in coordination with flagellar expression to achieve a successful UTI. In addition, inactivation of the RcsCDB system has been shown to decrease the ability of ExPEC resistance to serum-mediated killing [76]. This finding suggest that a tunable RcsCDB system may also be essential for the full virulence of ExPEC. Thus, hindering the proper function of the σ^E^ and RcsCDB signaling systems would be a potential strategy to fight against the bacterial infections.

The decreased motility may not be fully responsible for the defective ability of the ExPEC *prc* mutant to cause UTIs. Our previous study demonstrated that *prc* deletion decreases the resistance of ExPEC to complement-mediated killing in the bloodstream [4]. It has been suggested that pathogenic *E. coli* could be opsonized by the complement system in the UT [77]. Since bacterial opsonization could facilitate phagocytosis by phagocytes, leading to the elimination of invading bacteria in the tissue, defective resistance to the complement system may also attenuate the *prc* mutant in UTIs. In agreement with this hypothesis, we found that increasing the motility of Δ*prc*-RS218 by overexpressing *flhDC* to the level of *flhDC* expression in WT-RS218 could not restore the mutant’s UT colonization to the level of the wild-type strain (data not shown). On the other hand, the decreased motility was not responsible for the *prc* mutant’s defect in the resistance to killing mediated by the complement system because increasing the motility of Δ*prc*-RS218 did not restore bacterial resistance to complement-mediated serum killing (data not shown).

In addition to FliC, several other outer membrane proteins whose expression levels were significantly affected by the *prc* deletion were found in the LC/MS/MS analysis (Table 2). The altered expression levels of the proteins may also contribute the attenuated virulence of the ExPEC *prc* mutant. For example, SlyB was shown to be downregulated in the *prc* mutant of RS218 (Table 2). It is known that deletion of SlyB in *Burkholderia multivorans* attenuates bacterial iron uptake ability and compromises the OM integrity [78]. Given that the iron uptake ability and an intact OM integrity are important for pathogenic bacteria to invade hosts [79–81], it is worth further investigating whether the downregulated SlyB expression in the ExPEC *prc* mutant decreased the bacterial ability to cause UTIs. In addition, Tsx was shown to be upregulated in the *prc* mutant of RS218. The outer membrane porin Tsx is essential for *E. coli* to uptake of a gyrase inhibitor antibiotic, albicidin [82–84]. It has been known that deletion of *prc* increases the susceptibilities of *E. coli* to multiple antibiotics [85]. The increased Tsx level may be one of the contributing factors.

## Conclusions

In addition to our previous finding that lacking of Prc resulted in decreased ability of ExPEC to cause bacteremia [4], we demonstrated herein that such defect can also diminish the ability of ExPEC to cause UTIs. Given *prc* contributes to ExPEC infections in different extraintestinal host tissues as found in the bloodstream and UT, our study strengthens an idea that Prc or Prc homologs may be a potential antimicrobial target for developing a novel strategy in managing ExPEC or other bacterial infections. In addition, the σ^E^ and RcsCDB systems, which are responsible for the defect of the *prc* mutant in causing UTIs, are potential antimicrobial targets in the same light.

## Supplementary information

**Additional file 1: **T**able S1.** Primers used in this study

**Additional file 2: Table S2.** Identification of altered total proteins by liquid chromatography-tandem mass spectrometry

**Additional file 3: Table S3.** The differentially expressed genes in Δ*prc*-RS218 compared to those in WT-RS218 by RNA-seq

**Additional file 4: Figure S1.** The effects of overexpression of DegQ on motility, FliC expression, and activation of σ^E^ signaling. (**a**) Motility diameter of WT-RS218/pACYC184, Δ*prc*-RS218/pACYC184, and Δ*prc*-RS218/pDegQ. (**b**) FliC levels in WT-RS218/pACYC184, Δ*prc*-RS218/pACYC184, and Δ*prc*-RS218/pDegQ. (**c**) Promoter activity of *degP* in WT-RS218/pACYC184, Δ*prc*-RS218/pACYC184, and Δ*prc*-RS218/pDegQ. *degP* is positively regulated by the σ^E^ signaling system. The promoter activity of *degP* can reflect the activation level of σ^E^ signaling

## Data Availability

All data and materials are fully available without restriction and are within the manuscript and its Additional files.

## Abbreviations

2CSTSs: Two-component signal transduction systems
CFU: Colony-Forming unit
co-RNAP: core RNA polymerase
ESSSs: Extracytoplasmic stress signaling systems
EtBr: Ethidium bromide
ExPEC: Extraintestinal pathogenic *E. coli*
FPKM: Fragments per kilobase of transcript per million mapped reads
holo-RNAP: RNA polymerase holoenzyme
hpi: Hour post-inoculation
IACUC: Institutional Animal Care and Use Committee
IM: Inner membrane
LB: Luria Bertani
LC/MS/MS: Liquid chromatography-tandem mass spectrometry
M-MLV: Moloney murine leukemia virus
OM: Outer membrane
OMPs: OM-associated proteins
PCR: Polymerase chain reaction
qPCR: Real-time quantitative PCR
RNA-seq: RNA sequencing
RT-PCR: Reverse transcription-PCR
UT: Urinary tract
UTIs: Urinary tract infections
