# Supplementary material for: The role of the bacterial protease Prc in the uropathogenesis of extraintestinal pathogenic Escherichia coli
Source: J Biomed Sci. 2020 Jan 3;27:14. doi: 10.1186/s12929-019-0605-y (PMC6941253; doi:10.1186/s12929-019-0605-y)
Supplement: Supplementary file 1 — Additional file 1: Table S1. Primers used in this study [file 12929_2019_605_MOESM1_ESM.pdf]

Table S1. Primers used in this study.

Primers	Sequence (5'→3')
**Gene knockout**	
NK-*lacZ*-F	GTCGTGACTGGGAAAACCCTGGCGTTACCCA ACTTAATCGCATATCAATATCCTCCTTAG
NK-*lacZ*-R	CAACTGGTAATGGTAGCGACCGGCGCTCAGCT GGAATTCCGTGTAGGCTGGAGCTGCTTC
NK-*rseA*-F	CTTTAATGGATGGCGAAACGCTGGATAGTGA GCTGCTTATAGGAATATCCTCCTTAGTTC
NK-*rseA*-R	TGCTGAGTTTGTGCCTGCTCAAACTGAAGCTG TTCAGATGTGTAGGCTGGAGCTGCTTCG
NK-*degS*-F	ACGACGCCGATCAGATCATCGTCGCCTTACA GGATGGAATAGGAATATCCTCCTTAGTTC
NK-*degS*-R	ATGGTGACCTGCAATGTTAACTGTTTATCGTC ACGCATTGTGTAGGCTGGAGCTGCTTCG
NK-*rcsB*-F	AACCTGTATCACACCCGATG
NK-*rcsB*-R	CAGATCAGTTGGGATCGTTG
NK-*ompR*-F	GAACGTTATCTCACCGAACAAGGCTTCCAGG TTCGAAGCGTCGCTATAGGAATATCCTCC
NK-*ompR*-R	CGGATGCGCCGGATCTTCTTCCACCATGCGGC GCAGACGCGAAATTGTGTAGGCTGGAGC
NK-*qseB*-F	CTTTGCGGATCTGTTTGACG
NK-*qseB*-R	TACGCCAGTCGCGTTTAGTG
NK-*cpxR*-F	AAGGAGCTGCTCGAGATGGAAGGCTTCAACG TGATTGTTGCCCACATAGGAATATCCTCC
NK-*cpxR*-R	ATCGATAGCGCGGTCGAAAGGCGTCAGGCGC TTGCCCAGCACTTCTGTGTAGGCTGGAGC
NK-*fliC*-F	CAGTCTGCGCTGTCGAGTTCTATCGAGCGTCT GTCTTCTGGCTTGATAGGAATATCCTCC
NK-*fliC*-R	CTGGATAATCTGCGCTTTCGACATGTTGGACA CTTCGGTCGCATATGTGTAGGCTGGAGC
NK-*spr*-F	TTGTCGTTAAGGACTTCAAGGGAAAACAAAC AACATGGTCCATATGAATATCCTCCTTAG
NK-*spr*-R	GAGAACCCGGCGTGCTTCGTTGTAACGCTTCT TCCAGTATGTGTAGGCTGGAGCTGCTTC
**Spr-3xFlag**	
Spr-3xFLAG-F	GAAGCGTTACAACGAAGCACGCCGGGTTCTC AGCCGCAGCGACTACAAAGACCATGACG

3xFLAG-M13F-New-P2-R	CGGAATAGGAACTAAGGAGGATATTCCTATGT AAAACGACGGCCAGTGAATTG
New-P2	ATAGGAATATCCTCCTTAGTTC
Spr-3xFLAG-R	CTCGTCAGGATAGCCAAGGGATTGCATCCAAA CGGTTTATGTGTAGGCTGGAGCTGCTTC
**Complementary *degS gene* at the *lacZ* gene chromosomal locus**	
FD-lacZ-F	CCGGAAGAGAGTCAATTCAG
LacI-500 R	CATATGATAAGCATCGCTGCTCATACCAAAGCC GTTGATGGGTGTCTGGTCAG
lacZ-degQ-F	CTTGATGTCTCTGACCAGACACCCATCAACCT GAAGCCAGCAACGATCAG
degS-New-P2-R	CGGAATAGGAACTAAGGAGGATATTCCTATTTA GTTGGTCGCCGGATATTC
New-P2	ATAGGAATATCCTCCTTAGTTC
141-30-2	AGACCAACTGGTAATGGTAG
**Complementary *rcsB gene* at the *lacZ* gene chromosomal locus**	
FD-lacZ-F	CCGGAAGAGAGTCAATTCAG
LacI-500 R	CATATGATAAGCATCGCTGCTCATACCAAAGCC GTTGATGGGTGTCTGGTCAG
lacZ-rcsB-F	CTTGATGTCTCTGACCAGACACCCATCAACTT GGTACACGCTACTCTGTG
rcsB-New-P2-R	CGGAATAGGAACTAAGGAGGATATTCCTATTTA GTCTTTATCTGCCGGAC
New-P2	ATAGGAATATCCTCCTTAGTTC
141-30-2	AGACCAACTGGTAATGGTAG
degP-lacZ	
check-no lacI-F	GCTGTGCAACACAATACTGC
no lacI-UP500-R	TGACGCCGAAGTGAGATTTAAAATGCTGAC
no lacIp-Cm-F	GACCTGGCGTCAGCATTTTAAATCTCACTTCG GCGTCATGTGTAGGCTGGAGCTGCTTCG
NEW-P2	ATAGGAATATCCTCCTTAGTTC
Cm-degPp-F	CGGAATAGGAACTAAGGAGGATATTCCTATT AGCCATCCAGATGTCGAGCAGCTTG
Cm-degPp-R	TCCTGTGTGAAATTGTTATTCGCTCACAATCTC AGAGCCAGTGCACTCAGTGCTAATGTG
lacZ-500-F	ATTGTGAGCGAATAACAATTTCACACAG
lacZ-500-R	ACCACAGATGAAACGCCGAG
**qPCR or RT-PCR**	

ftsZ-RT-F	CAATGGAACTTACCAATGAC
ftsZ-RT-R	TGTTTTACGCAGCGCTTGTG
flhD-RT-F	TCCGCTATGTTTCGTCTCGGCATA
flhD-RT-R	ACCAGTTGATTGGTTTCTGCCAGC
fliA-RT-F	AACGCTATGACGCCCTACAAGGAA
fliA-RT-R	AGTTCCTGCTCCAGTTGCCCTATT
flgE-RT-F	CACGTTTAGCCTGAGCTTCC
flgE-RT-R	CAACCGTACCGTCATCATTG
flhA-RT-F	ACGAGAAACCGACCCATGAG
flhA-RT-R	CCATCATCGACAAGATCAAC
fliF-RT-F	AATGCGACTGCAGCCCAGAC
fliF-RT-R	AGGATCAGTGCGACCATGAC
fliM-RT-F	GATAACGACATGGGCGATAG
fliM-RT-R	CACTTTCGCCGCTAACACTG
fliE-RT-F	GTTATCAGCCAGTTACAGGC
fliE-RT-R	TGTGTATCGCTTATGCGATC
fliT-RT-F	ATTTCGCCTGGCAACAACTC
fliT-RT-R	TGCACCGCATTCACATACGC
flgM-RT-F	GAGTATTGATCGCACTTCGC
flgM-RT-R	ACGTCACACTGGTGCTGGTG
fliC-RT-F	ACAGCCTCTCGCTGATCACTCAA
fliC-RT-R	GCGCTGTTAATACGCAAGCCAGA
motA-RT-F	GCGATTAAAGGCACGCTGAAGG
motA-RT-R	GAAGGTGTTCATGTGACCGCTG
tar-RT-F	TTAACCTGAGTCGTTCAGCG
tar-RT-R	TTTCAGGTAACGGTGCCATG
yiiS-RT-F	CGACAGAGAGGCGAATATACAGAGGTGCCC
yiiS-RT-R	CTCGGCCTGGCATGAGAAAGTGAAGGCCGC
hpf-RT-F	ATAACGTCGAGATCACCGAG
hpf-RT-R	ATGTTTGGTCAACTGGCGTG
fkpA-RT-F	CACTTTTGCTGCTGAAGCTG
fkpA-RT-R	GTCTGTTCGATCTCTTGGTC
yjbE-RT-F	TTTGCCATATCTGCGCTTG
yjbE-RT-R	TGGTGGTCCCGGTATTAGAA
osmC-RT-F	CCATAAGAAAGGTCAGGCAC
osmC-RT-R	TGCGCCAATCAGTTCTTCAG
aroG-RT-F	TGGGCTGGAAAGGGCTGATT
aroG-RT-R	GAGAAACTCACCCGCCGCTG

mviM-RT-F	TGACTGGACGTTACAAGGTG
mviM-RT-R	AGTAACGTACTGACCACGTC
qseB-RT-F	CCTTATGATGCGGTGATCCTGG
qseB-RT-R	TCCCAGACGCAGCCCTTCTA
ompC-RT-F	TCCTGGTCCCAGCTCTGCTG
ompC-RT-R	CCAAGACGCATGTAGGTCTG
**Plasmid pPrc-S430A**	
prcS430A-F	GTCCCGGGCGTGCCCTGGTTGTGGGTG
prcS430A-R	GCTACGTAATCCTGCATTGCCGCGGCAAAGATTTCTGAAGCGGCAGCACTGAAGCGGTCA
**Plasmid pPrc-K455A**	
prcK455A-F	CACCCGGGCGTGCCCTGGTTGTGGGTGAACCGACATTTGGTGCCGGCACCGTTCAGCAGT
prcK455A-R	CGTACGTAATCCTGCATTGCCGCGGC
**Plasmid pDegQ**	
pACYC184-CmP-degQ-F	TCGAGATTTTCAGGAGCTAAGGAAGCTAAATTGAATCTCTTTTCTTATCATTCAGGTACG
degQ-NotI-R	ATATGCGGCCGCTTAACGCATTAGTAGGTAGAG
Cm-promoter-F	TTTAGCTTCCTTAGCTCCTG
Cm-out-R	AGTCGCGGCCGCATACGCAAGGCGACAAGGTG
**Plasmid pFlhDC**	
pUC19-HA tag-flhD-HindIII-F	CGCCAAGCTTGTATCCATATGATGTTCCAGATTATGCTGTGGGAATAATGCATACCTC
pUC19-flhC-his tag-BamHI-R	CCGGGGATCCTTAATGATGATGATGATGATGAACAGCCTGTACTCTCTGTTC
**Plasmid pBAD-FlhDC**	
pBAD-HA-flhDC-NcoI-F	TTAACCATGGATTATCCATATGATGTTCCAGATTATGCTGTGGGAATAATGCATACCTC
pUC19-flhC-his tag-BamHI-R	CCGGGGATCCTTAATGATGATGATGATGATGAACAGCCTGTACTCTCTGTTC
**Plasmid pRcsB**	
pBAD-RcsB-NcoI-F	TTAACCATGGCAGTTATGTCAAGAGC
pBAD-RcsB-EcoRI-R	CTTCGAATTCTTAGTCTTTATCTGCCGGAC
**Plasmid pRseA**	
pBAD-HA-rseA-NcoI-F	TTAACCATGGATTATCCATATGATGTTCCAGATTATGCTATGCAGAAAGAACAACTTTC

pBAD-rseA-his tag-HindIII-R	ATATAAGCTTTTAGTGATGGTGATGGTGATGC TGCGATTGCGTTCCTAAAG
**Plasmid pSpr**	
pBAD-NcoI-spr-F	TTAACCATGGTCAAATCTCAACCG
Spr-Flag-BamHI-R	CTTCGGATCCGAATTCACTTGTCGTCATCGTC TTTGTAGTCGCTGCGGCTGAGAACCCG
