# Supplementary material for: The role of the bacterial protease Prc in the uropathogenesis of extraintestinal pathogenic Escherichia coli
Source: J Biomed Sci. 2020 Jan 3;27:14. doi: 10.1186/s12929-019-0605-y (PMC6941253; doi:10.1186/s12929-019-0605-y)
Supplement: Supplementary file 2 — Additional file 2: Table S2. Identification of altered total proteins by liquid chromatography-tandem mass spectrometry [file 12929_2019_605_MOESM2_ESM.pdf]

Table S2. Identification of altered total proteins by liquid chromatography-tandem mass spectrometry.

Protein name	Description	Fold Change*	P value
**Downregulated protein in Δ*prc*-RS218**			
K3J1Q6	Uncharacterized protein	(-)100	3.12E-02
DctA	C4-dicarboxylate transport protein	(-)100	1.75E-02
NarG	Nitrate reductase, alpha subunit	(-)100	1.59E-02
FadD	Long-chain-fatty-acid--CoA ligase	(-)100	1.15E-02
GrxB	Glutaredoxin-2	(-)100	7.30E-03
BglA	6-phospho-beta-glucosidase BglA	(-)100	5.49E-03
M8NDU5	Glycosyl hydrolase 1 family protein	(-)100	5.49E-03
GyrB	DNA gyrase subunit B	(-)100	2.31E-03
F1ZQG5	Putative uncharacterized protein	(-)100	1.62E-03
PepB	Peptidase B	(-)100	1.09E-03
SlyB	Outer membrane lipoprotein SlyB	(-)100	5.30E-04
PrfC	Peptide chain release factor 3	(-)100	4.10E-04
FliC	Flagellin	(-)100	2.50E-04
AtpA	ATP synthase subunit alpha	(-)100	1.60E-04
FadE	Acyl-CoA dehydrogenase	(-)100	5.00E-05
RfaE	Heptose 1-phosphate adenyltransferase	(-)6.37	4.78E-02
RplA	50S ribosomal protein L1	(-)5.12	2.93E-02
FdhE	Formate dehydrogenase formation protein	(-)5	4.11E-02
ProX	Glycine betaine/proline ABC transporter - periplasmic binding protein	(-)4.75	2.82E-02
DapA	4-hydroxy-tetrahydrodipicolinate synthase	(-)4.6	4.27E-02
Dxs	1-deoxy-D-xylulose-5-phosphate synthase	(-)4.45	4.34E-02
NuoC	NADH-quinone oxidoreductase subunit C/D	(-)3.99	4.64E-02
EF-Tu	Elongation factor Tu 2	(-)2.71	5.70E-04
GdhA	Glutamate dehydrogenase	(-)2.28	2.35E-02
B3Y1K2	AidA-I adhesin-like protein	(-)2.06	3.51E-02
**Upregulated protein in Δ*prc*-RS218**			
AsnS	Asparagine--tRNA ligase	(+)100	3.44E-02

ArgT	Lysine-arginine-ornithine-binding periplasmic protein	(+)100	1.98E-02
MetQ	Methionine ABC transporter substrate-binding protein	(+)100	1.05E-02
Hfq	RNA-binding protein Hfq	(+)100	7.90E-03
W1D8I5	Dienelactone hydrolase and related enzymes	(+)100	6.82E-03
ThrC	Threonine synthase	(+)100	3.29E-03
AphA	Class B acid phosphatase	(+)100	3.26E-03
IcdA	Isocitrate dehydrogenase	(+)100	2.70E-03
FkpA	FKBP-type peptidyl-prolyl cis-trans isomerase	(+)100	1.13E-03
Spr	Murein DD-endopeptidase	(+)100	1.10E-03
YraP	Phospholipid-binding domain protein	(+)100	8.40E-04
NanA	N-acetylneuraminate lyase	(+)100	7.80E-04
Ag43	Antigen 43	(+)100	7.80E-04
GlgC	Glucose-1-phosphate adenylyltransferase	(+)100	5.10E-04
FumA	Fumarate hydratase class I, aerobic	(+)100	3.40E-04
BamA	Outer membrane protein assembly factor	(+)100	3.30E-04
FabD	Malonyl CoA-acyl carrier protein transacylase	(+)100	2.40E-04
HsdR	Type I restriction enzyme EcoKI R protein	(+)100	1.40E-04
V6FBZ4	PF12082 domain protein	(+)100	1.40E-04
ArgF	Ornithine carbamoyltransferase	(+)100	1.10E-04
GlmS	Glutamine--fructose-6-phosphate aminotransferase	(+)100	6.00E-05
GudD	Glucarate dehydratase	(+)100	1.00E-05
Tsx	Nucleoside-specific channel-forming protein	(+)100	9.78E-07
DadA	D-amino acid dehydrogenase small subunit	(+)5.07	2.98E-02
MinD	Septum site-determining protein MinD	(+)3.34	1.16E-02
TraT	Conjugal transfer surface exclusion protein TraT	(+)3.17	1.55E-02
KatG	Catalase-peroxidase	(+)2.94	2.91E-02

HisG	ATP phosphoribosyltransferase	(+)2.71	4.17E-02
ThrA	Aspartokinase I-homoserine dehydrogenase I	(+)2.7	6.90E-04
L3TFU9	ChvD family ATP-binding cassette protein	(+)2.38	2.87E-03
TolC	Outer membrane channel protein TolC	(+)2.21	4.47E-03
NagA	N-acetylneuraminate synthase	(+)2.12	9.40E-04

*** (-)**, indicates that the protein was downregulated in Δ*prc*-RS218 compared to in WT-RS218.

(+), indicates that the protein was upregulated in Δ*prc*-RS218 compared to in WT-RS218.
