# Supplementary material for: The role of the bacterial protease Prc in the uropathogenesis of extraintestinal pathogenic Escherichia coli
Source: J Biomed Sci. 2020 Jan 3;27:14. doi: 10.1186/s12929-019-0605-y (PMC6941253; doi:10.1186/s12929-019-0605-y)
Supplement: Supplementary file 3 — Additional file 3: Table S3. The differentially expressed genes in Δprc-RS218 compared to those in WT-RS218 by RNA-seq [file 12929_2019_605_MOESM3_ESM.pdf]

Table S3. The differentially expressed genes in Δ*prc* -RS218 compared to those in WT-RS218 by RNA-seq.

contig	RS218 locus_tag	Gene name	Gene product / functional description	Fold change	P value
g100.t1	W817_00215	*C1129*	ligand-gated channel	0.46	0.042
g1000.t1	W817_02290	*prpR*	propionate catabolism operon regulatory protein PrpR	3.93	0.006
g1010.t1	W817_02335	*lacZ*	beta-galactosidase	0.48	0.011
g1013.t1	W817_02345	*yaiL*	nucleoprotein/polynucleotide-associated enzyme	1.42	0.047
g1015.t1	W817_02350	*yaiM*	S-formylglutathione hydrolase	0.28	0.012
g102.t1	W817_00230	*int*	recombinase	0.32	0.041
g1020.t1	W817_02375	*yaiP*	membrane protein	0.03	0.033
g103.t1	W817_00235	*P050*	RepFIB replication protein A	0.41	0.014
g1035.t1	W817_02440	*yaiZ*	hypothetical protein	2.88	0.007
g1037.t1	W817_02455	*iraP*	anti-RssB factor	2.79	0.011
g1046.t1	W817_02500	*yaiE*	hypothetical protein	2.46	0.012
g1066.t1	W817_02595	*yajD*	hypothetical protein	2.16	0.03
g1068.t1	W817_02605	*yajI*	hypothetical protein	2.4	0.013
g1069.t1	W817_02610	*ybaD*	NrdR family transcriptional regulator	2.13	0.027
g1075.t1	W817_02640	*yajO*	oxidoreductase	2.49	0.042
g1079.t1	W817_02660	*thiI*	tRNA s(4)U8 sulfurtransferase	0.43	0.008
g1080.t1	W817_02665	*thiJ*	oxidative-stress-resistance chaperone	1.49	0.009
g1092.t1	W817_02720	*bolA*	BolA family transcriptional regulator	3.85	0.04
g1095.t1	W817_02740	*clpX*	ATPase subunit of ClpXP protease	1.43	0.034
g1104.t1	W817_02775	*ybaE*	hypothetical protein	2.78	0.001
g1106.t1	W817_02785	*ybaO*	hypothetical protein	0.37	0.033
g1115.t1	W817_02830	*ylaB*	hypothetical protein	0.51	0.008
g1117.t1	W817_02840	*maa*	maltose O-acetyltransferase	0.13	0.017
g1131.t1	W817_02890	*apt*	adenine phosphoribosyltransferase	0.5	0.022
g115.t1	W817_00305	*P066*	hypothetical protein	0.37	0.049
g1151.t1	W817_02980	*copA*	copper exporting ATPase	0.46	0.039
g1152.t1	W817_02985	*ybaS*	glutaminase	0.44	0.007
g1156.t1	W817_03005	*C0520*	hypothetical protein	0.34	0.012
g1157.t1	W817_03010	*C0521*	adhesin	0.41	0.045
g1158.t1	W817_03015	*C0522*	hypothetical protein	0.47	0.035
g116.t1	W817_00310	*P067*	transposase	0.07	0.029
g117.t1	W817_00315	*yccB*	hypothetical protein	0.39	0.012
g1170.t1	W817_03070	*ybbS*	LysR family transcriptional regulator	0.48	0.006
g1174.t1	W817_03090	*hyi*	hydroxypyruvate isomerase	0.34	0.002
g118.t1	W817_00320	*istB*	ATPase AAA	2.37	0.035
g1190.t1	W817_03175	*ppiB*	peptidyl-prolyl cis-trans isomerase B	1.89	0.018
g1195.t1	W817_03210	*C0563*	integrase	0.31	0.012
g1196.t1	W817_03215	*tfaQ*	tail fiber assembly protein	0.26	0.013
g120.t1	W817_00330	*P073*	hypothetical protein	3.33	0.046
g1201.t1	W817_03245	*cusS*	sensor kinase CusS	0.49	0.01
g1205.t1	W817_03260	*cusF*	copper-binding protein	0.4	0.031
g1209.t1	W817_03280	*ybdG*	miniconductance mechanosensitive channel	1.14	0.038
g1215.t1	W817_03320	*fepA*	outer membrane receptor FepA	0.23	0.024
g1218.t1	W817_03335	*entF*	enterobactin synthase subunit F	0.43	0.043
g1221.t1	W817_03345	*fepC*	ferric enterobactin transport ATP-binding protein fepC	0.42	0.04
g1223.t1	W817_03350	*fepG*	iron-enterobactin transporter permease	0.59	0.038
g1227.t1	W817_03370	*entC*	isochorismate synthase EntC	0.51	0.016
g1228.t1	W817_03375	*entE*	enterobactin synthase subunit E	0.3	0.035
g1234.t1	W817_03400	*ybdD*	hypothetical protein	2.43	0.028
g1236.t1	W817_03410	*ybdL*	aminotransferase	0.2	0.004
g1237.t1	W817_03415	*ybdM*	hypothetical protein	0.18	0.012
g1238.t1	W817_03420	*ybdN*	hypothetical protein	0.15	0.017
g1245.t1	W817_03450	*uspG*	universal stress protein G	2.54	0.038
g1255.t1	W817_03505	*dpiB*	sensor histidine kinase DpiB	0.28	0.049
g1256.t1	W817_03510	*dpiA*	two-component response regulator DpiA	0.43	0.019
g1278.t1	W817_03615	*ybeL*	hypothetical protein	2.88	0.025
g1299.t1	W817_03745	*nagE*	N-acetylglucosamine PTS permease	1.82	0.044
g1305.t1	W817_03765	*terC*	tellurium resistance protein TerC	0.12	0.008
g1316.t1	W817_03820	*fldA*	flavodoxin	2.32	0.001
g1342.t1	W817_03945	*sdhC*	succinate dehydrogenase cytochrome b-556	2.04	0.006
g1352.t1	W817_03985	*C0725*	hypothetical protein	0.16	0.012
g1362.t1	W817_04035	*tolB*	the Tol-Pal cell envelope complex	1.44	0.025

g1369.t1	W817_04105	*aroG*	2-dehydro-3-deoxyphosphoheptonate aldolase	1.93	0.004
g1378.t1	W817_04150	*modA*	molybdenum ABC transporter substrate-binding protein	2.75	0.027
g1388.t1	W817_04185	*ybhI*	membrane protein	0.27	0.008
g1417.t1	W817_04310	*ybhG*	transporter	2.93	0.021
g1418.t1	W817_04315	*ybiH*	transcriptional regulator	2.87	0.032
g142.t1	W817_22780	*C5110*	DNA adenine methylase	0.42	0.034
g1432.t1	W817_04390	*glnP*	glutamine ABC transporter permease	2.38	0.003
g1433.t1	W817_04395	*glnH*	amino acid ABC transporter substrate-binding protein	3.57	0.03
g1437.t1	W817_04415	*ybiP*	phosphoethanolamine transferase	0.35	0.002
g1442.t1	W817_04440	*ybiT*	heme ABC transporter ATP-binding protein	0.45	0.036
g1448.t1	W817_04475	*moeA*	molybdopterin biosynthesis protein MoeA	2.35	0.007
g1449.t1	W817_04480	*iaaA*	isoaspartyl peptidase	2.4	0.001
g1457.t1	W817_04515	*rimO*	30S ribosomal protein S12 methylthiotransferase	0.39	0.003
g1458.t1	W817_04520	*yliH*	biofilm formation regulatory protein BssR	2.11	0.02
g1459.t1	W817_04525	*yliI*	aldose dehydrogenase	0.45	0.043
g1461.t1	W817_04535	*dacC*	penicillin-binding protein 6	1.33	0.015
g1472.t1	W817_04605		hypothetical protein	0	0.006
g1475.t1	W817_04620	*dam*	DNA adenine methylase	0.14	0.031
g1505.t1	W817_04775		tail sheath protein	0.3	0.014
g1512.t1	W817_04815	*ybjL*	transporter	0.51	0.026
g1525.t1	W817_04880	*artJ*	arginine ABC transporter substrate-binding protein	0.51	0.034
g1544.t1	W817_04980	*macA*	macrolide transporter subunit	1.43	0.002
g1546.t1	W817_04990	*cspD*	cold-shock protein	7.07	0.039
g1548.t1	W817_05000	*clpA*	ATP-dependent Clp protease ATP-binding protein	3.29	0.031
g1557.t1	W817_05045	*aat*	leucyl/phenylalanyl-tRNA--protein transferase	0.46	0.041
g1564.t1	W817_05080	*rarA*	recombinase RarA	1.39	0.006
g157.t1	W817_22865	*C5124*	tail protein	2.99	0.047
g158.t1	W817_22870	*C5125*	tail protein	2.59	0.02
g1580.t1	W817_05150	*ycaL*	metalloprotease	0.6	0.017
g1584.t1	W817_05165	*ihfB*	integration host factor subunit beta	1.98	0.017
g1610.t1	W817_05270	*pepN*	aminopeptidase N	2.13	0.006
g1611.t1	W817_05275	*ssuB*	aliphatic sulfonate ABC transporter ATP-binding protein	0.46	0.012
g1612.t1	W817_05280	*ssuC*	sulfonate ABC transporter	0.16	0.004
g1613.t1	W817_05285	*ycbN*	alkanesulfonate monooxygenase	0.06	0.005
g1634.t1	W817_05395	*mgsA*	methylglyoxal synthase	2.51	0.003
g1635.t1	W817_05400	*yccT*	hypothetical protein	0.39	0.003
g1636.t1	W817_05405	*C1031*	hypothetical protein	2.47	0.028
g1656.t1	W817_05480	*appA*	periplasmic AppA protein	0.44	0.046
g1660.t1	W817_05505	*yccM*	electron transporter YccM	0.38	0.017
g1670.t1	W817_05550	*agp*	glucose-1-phosphatase/inositol phosphatase	3.54	0.039
g1671.t1	W817_05555	*yccJ*	hypothetical protein	2.16	0.018
g1679.t1	W817_05590	*ycdK*	aminoacrylate peracid reductase	2.2	0.029
g1682.t1	W817_05605	*rutR*	TetR/AcrR family transcriptional regulator	1.41	0.029
g1686.t1	W817_05610	*putA*	proline dehydrogenase	3.45	0.013
g1687.t1	W817_05620	*putP*	proline:sodium symporter PutP	3.22	0.001
g1691.t1	W817_05630	*ycdO*	iron ABC transporter substrate-binding protein	0.42	0.002
g1692.t1	W817_05635	*ycdB*	peroxidase	0.41	0.017
g1696.t1	W817_05640	*phoH*	phoH-like family protein	2.29	0.013
g1698.t1	W817_05650	*ycdQ*	N-glycosyltransferase	0.26	0.042
g1700.t1	W817_05660	*pgaA*	poly-beta-1,6-N-acetyl-D-glucosamine export protein	0.36	0.024
g1701.t1	W817_05665	*ycdT*	hypothetical protein	0.29	0.038
g1715.t1	W817_05760	*sfaC*	putative S fimbrial switch regulatory protein	2.33	0.021
g1717.t1	W817_05780	*sfaE*	S fimbriae periplasmic chaperone SfaE	0.45	0.028
g1723.t1	W817_05810	*iroN*	outer membrane receptor IroN	0.16	0.012
g1724.t1	W817_05815	*iroE*	IroE protein	0.16	0.038
g1730.t1	W817_05830	*iroB*	glucosyltransferase	0.4	0.001
g1735.t1	W817_05880	*C1131*	cobalamin biosynthesis protein CobW	0.16	0.013
g1736.t1	W817_05885	*C1132*	transposase	0.09	0.042
g1738.t1	W817_05905	*C1137*	hypothetical protein	0.42	0.038
g1739.t1	W817_05910		hypothetical protein	0	0.009
g174.t1	W817_22955	*qor*	quinone oxidoreductase	2.22	0.027
g1768.t1	W817_06065	*mdoG*	periplasmic glucan biosynthesis	1.37	0.006
g178.t1	W817_22975	*C4623*	CoA-transferase	0.5	0.036
g1785.t1	W817_06155	*rimJ*	alanine acetyltransferase	2.72	0.035

g1786.t1	W817_06160	*yceH*	hypothetical protein	2.34	0.04
g1787.t1	W817_06165	*mviM*	putative virulence factor	2.09	0.016
g183.t1	W817_22995	*C4627*	nicotinamide mononucleotide transporter	0.41	0.017
g1836.t1	W817_06375	*ycfJ*	hypothetical protein V413_00470	3.15	0.022
g1849.t1	W817_06430	*ycfZ*	membrane protein	0.43	0.046
g1850.t1	W817_06435	*ymfA*	membrane protein	0.15	0.005
g1854.t1	W817_06455	*potA*	putrescine/spermidine ABC transporter ATPase	0.47	0.033
g1867.t1	W817_06520	*C1269*	hypothetical protein	0.05	0.043
g1868.t1	W817_06525	*C1271*	RNA-binding protein	0.18	0.025
g1869.t1	W817_06550	*C1275*	exonuclease	0.27	0.038
g1885.t1	W817_06705		Rz endopeptidase	0.33	0.024
g1888.t1	W817_06730	*ybcX*	terminase	0.5	0.028
g1893.t1	W817_06745	*C1315*	scaffold protein	1.76	0.038
g1917.t1	W817_06855	*sitA*	iron ABC transporter substrate-binding protein	0.47	0.018
g1920.t1	W817_06885	*ycgF*	blue light- and temperature-regulated antirepressor YcgF	0.28	0.042
g1922.t1	W817_06900	*ymgB*	two-component-system connector protein AriR	6.86	0.031
g1925.t1	W817_15550	*C1350*	transposase IS1	0.5	0.008
g1927.t1	W817_06925	*C1353*	hypothetical protein	0.07	0.011
g1928.t1	W817_06930	*ymgD*	hypothetical protein	0.22	0.041
g1929.t1	W817_06935	*ymgG*	hypothetical protein	0.51	0.03
g1930.t1	W817_06940	*C1357*	ATPase	0.42	0.016
g1935.t1	W817_06965	*ycgK*	protein YcgK	2.91	0.014
g1939.t1	W817_06985	*C1368*	hemolysin activation protein HecB	0.26	0.046
g1944.t1	W817_07010	*fadR*	fatty acid metabolism regulator	2.17	0.007
g1946.t1	W817_07020	*dadA*	D-amino acid dehydrogenase small subunit	2.67	0.017
g1950.t1	W817_07030	*cvrA*	cell volume regulation protein A	0.42	0.003
g1961.t1	W817_07085	*treA*	trehalase	0.32	0.004
g1969.t1	W817_07125	*ychH*	membrane protein	4.8	0.015
g1974.t1	W817_07145	*hemM*	outer membrane lipoprotein LolB	1.87	0.01
g1981.t1	W817_07175	*kdsA*	2-dehydro-3-deoxyphosphooctonate aldolase	1.43	0.045
g1983.t1	W817_07185	*chaA*	sodium:proton antiporter	0.44	0.019
g2025.t1	W817_07380	*ompW*	outer membrane protein W	2.78	0.03
g2044.t1	W817_07525	*C1490*	lysozyme	0.26	0.014
g2057.t1	W817_07585	*C1504*	tail attachment protein	2.71	0.036
g2065.t1	W817_07635	*C1512*	tail protein	0.35	0.035
g2079.t1	W817_07705	*trpA*	tryptophan synthase subunit alpha	6.33	0.015
g2080.t1	W817_07710	*trpB*	tryptophan synthase subunit beta	5.42	0.027
g2082.t1	W817_07715	*trpC*	phosphoribosylanthranilate isomerase	7.1	0.011
g2084.t1	W817_07720	*trpD*	anthranilate phosphoribosyltransferase	4.24	0.01
g2092.t1	W817_07755	*yciK*	oxoacyl-ACP reductase	2.35	0.003
g2093.t1	W817_07760	*sohB*	serine protease SohB	1.51	0.027
g2098.t1	W817_07785	*C1546*	hypothetical protein	0.34	0.017
g2100.t1	W817_07795	*acnA*	aconitate hydratase 1	2.98	0.041
g2101.t1	W817_07800	*ribA*	GTP cyclohydrolase II	2.1	0.009
g2107.t1	W817_07830	*osmB*	osmotically inducible lipoprotein	1.9	0.048
g2111.t1	W817_07850	*rnb*	exoribonuclease II	0.66	0.021
g2112.t1	W817_07855	*yciW*	hypothetical protein	2.12	0.001
g2130.t1	W817_07945	*pspE*	thiosulfate:cyanide sulfurtransferase	3.11	0.026
g2133.t1	W817_07960	*ycjO*	sugar ABC transporter permease	0.36	0.02
g2142.t1	W817_08000	*ompG*	membrane protein	0.24	0.029
g2147.t1	W817_08025	*tpx*	lipid hydroperoxide peroxidase	2.1	0.037
g2156.t1	W817_08070	*uspE*	universal stress protein E	2.5	0.039
g2157.t1	W817_08075	*fnr*	fumarate/nitrate reduction regulatory protein	1.28	0.03
g2169.t1	W817_08145	*uspF*	universal stress protein F	4.68	0.024
g2177.t1	W817_08165	*hslJ*	heat shock protein	1.71	0.033
g2189.t1	W817_08200	*ynbA*	membrane protein	0.26	0.022
g2205.t1	W817_08275	*mdoD*	glucan biosynthesis protein D	1.52	0.03
g2206.t1	W817_08280	*ydcH*	hypothetical protein	4.24	0.028
g2208.t1	W817_08290	*ydcK*	acetyltransferase	0.38	0.029
g2214.t1	W817_08320	*ydcP*	predicted peptidase	0.7	0.017
g2255.t1	W817_08530	*rpsV*	30S ribosomal protein S22	2.58	0.018
g2257.t1	W817_08540	*osmC*	peroxiredoxin	2.32	0.035
g2258.t1	W817_08545	*dosP*	diguanylate phosphodiesterase	0.43	0.025
g2266.t1	W817_08590	*ydeM*	anaerobic sulfatase maturase	0.24	0.025

g2321.t1	W817_08880	*rspB*	starvation sensing protein RspB	0.15	0.035
g2322.t1	W817_08885	*rspA*	bifunctional D-altronate/D-mannonate dehydratase	0.36	0.007
g2333.t1	W817_08930	*ynfH*	dimethyl sulfoxide reductase subunit C	0.22	0.012
g2342.t1	W817_08975	*ydgD*	serine protease	0.6	0.01
g2349.t1	W817_09010	*ydgI*	arginine:ornithine antiporter	0.38	0.02
g2358.t1	W817_09045	*fumA*	fumarate hydratase	2.19	0.021
g237.t1	W817_23245	*yjcT*	D-allose kinase	0.5	0.035
g2374.t1	W817_09100	*add*	adenosine deaminase	2.25	0.019
g2379.t1	W817_09120	*rnfA*	electron transport complex RsxE subunit	0.49	0.048
g2383.t1	W817_09130	*rnfC*	electron transporter RnfC	0.31	0.013
g2385.t1	W817_09135	*rnfD*	electron transporter RnfG	0.54	0.047
g2387.t1	W817_09140	*RsxA*	electron transport complex protein RsxA	0.21	0.032
g2400.t1	W817_09195	*slyB*	outer membrane lipoprotein SlyB	1.81	0.012
g2404.t1	W817_09215	*ydhK*	fusaric acid resistance protein	0.37	0.036
g2405.t1	W817_09220	*sodC*	superoxide dismutase	2.22	0.011
g2415.t1	W817_09265	*sodB*	superoxide dismutase	3.37	0.034
g242.t1	W817_23265	*yjcX*	D-allose-binding periplasmic protein precursor	0.32	0.016
g2429.t1	W817_09350	*ydhW*	hypothetical protein	0.32	0.023
g2432.t1	W817_09355	*ydhV*	oxidoreductase	0.49	0.023
g2455.t1	W817_09450	*ydiB*	shikimate 5-dehydrogenase	0.33	0.007
g2474.t1	W817_09535	*nlpC*	lipoprotein NlpC	1.9	0.017
g2478.t1	W817_09555	*ihfA*	integration host factor subunit alpha	2.47	0.039
g2486.t1	W817_09605	*ydiY*	membrane protein	0.41	0.013
g2489.t1	W817_09620	*yniA*	hypothetical protein	2.66	0.047
g25.t1	W817_00435	*yijE*	lytic transglycosylase	0.42	0.011
g2507.t1	W817_09700	*ydjR*	hypothetical protein	0.24	0.015
g2532.t1	W817_09810	*selD*	selenophosphate synthetase	2.46	0.019
g2533.t1	W817_09815	*ydjA*	oxidoreductase	2.15	0.039
g2534.t1	W817_09820	*sppA*	protease IV	1.38	0.034
g2539.t1	W817_09835	*ydjE*	MFS transporter	0.09	0.029
g2542.t1	W817_09850	*ydjH*	sugar kinase	0.44	0.031
g2587.t1	W817_10075		hypothetical protein	2.9	0.006
g2594.t1	W817_10120	*mor*	DNA-binding protein	2.54	0.026
g2596.t1	W817_10135	*ydjK*	inner membrane metabolite transport protein ydjK	0.4	0.004
g2598.t1	W817_10140	*ydjL*	alcohol dehydrogenase	0.39	0.001
g2599.t1	W817_10145	*yeaC*	hypothetical protein	2.11	0.001
g2600.t1	W817_10150	*msrB*	methionine sulfoxide reductase B	2.45	0.034
g2609.t1	W817_10180	*yeaH*	hypothetical protein	0.31	0.03
g2610.t1	W817_10185	*yeaI*	membrane protein	0.12	0.035
g2611.t1	W817_10190	*YeaJ*	diguanylate cylase	0.21	0.045
g2618.t1	W817_10220	*yeaO*	hypothetical protein	2.3	0.017
g2627.t1	W817_10265	*fadD*	long-chain-fatty-acid-CoA ligase	2.77	0.004
g2627.t1	W817_10265	*fadD*	long-chain-fatty-acid--CoA ligase	2.77	0.004
g2628.t1	W817_10270	*yeaY*	predicted lipoprotein	2.02	0.029
g2650.t1	W817_10385	*yebQ*	multidrug MFS transporter	0.45	0.002
g2652.t1	W817_10390	*htpX*	heat shock protein HtpX	1.79	0.013
g2654.t1	W817_10395	*prc*	carboxy-terminal protease	0	0.013
g2667.t1	W817_10455	*yobA*	hypothetical protein	2.18	0.029
g2673.t1	W817_10490	*yebF*	protein yebF	0.36	0.033
g27.t1	W817_00455	*traA*	conjugal transfer protein TraA	0.46	0.004
g2704.t1	W817_10635	*yecM*	hypothetical protein	1.61	0.005
g2731.t1	W817_10770	*ftnA*	ferritin	2.1	0.014
g2739.t1	W817_10825	*sdiA*	transcriptional regulator	0.32	0.007
g2747.t1	W817_10870	*fliD*	flagellar capping protein	0.31	0.011
g2754.t1	W817_10900	*yedF*	hypothetical protein	4.23	0.034
g2759.t1	W817_10935	*fliE*	flagellar hook-basal body protein FliE	0.46	0.003
g2778.t1	W817_11025	*yedQ*	putative diguanylate cyclase YedQ	0.7	0.042
g2792.t1	W817_11100	*yedZ*	sulfite oxidase subunit YedZ	0.45	0.028
g2795.t1	W817_11125	*C2177*	truncated integrase B	0.4	0.008
g28.t1	W817_00460	*traL*	conjugal transfer protein TraL	0.21	0.011
g2800.t1	W817_11150	*ybtP*	lipoinner membrane ABC-transporter domain protein	2.17	0.02
g2825.t1	W817_11250	*C2204*	integrase	0.29	0.018
g2826.t1	W817_11255	*C2206*	hypothetical protein	2.51	0.034
g285.t1	W817_23485	*yjdL*	peptide permease	0.15	0.007

g2853.t1	W817_11360	*C2226*	transposase	0.3	0.038
g2859.t1	W817_11395	*C2234*	Colicin I receptor precursor	0.48	0.006
g2865.t1	W817_11425	*C2241*	acetyltransferase	0.17	0.013
g2866.t1	W817_11430	*yaiO*	membrane protein	0.34	0.029
g287.t1	W817_23495	*cadB*	arginine:agmatine antiporter	0.25	0.047
g2870.t1	W817_11455	*C2245*	hypothetical protein	0.29	0.04
g2871.t1	W817_11460	*ydcZ*	membrane protein	0.31	0.01
g288.t1	W817_23500	*cadC*	transcriptional regulator	0.31	0.047
g2888.t1	W817_11575	*C2269*	chemotaxis protein	1.8	0.022
g2897.t1	W817_11620	*yeeV1*	toxin	0.31	0.008
g29.t1	W817_00465	*traE*	conjugal transfer protein TraE	0.28	0.035
g290.t1	W817_23515	*dsbD*	thiol:disulfide interchange protein DsbD	1.28	0.012
g2901.t1	W817_11650	*sbmC*	DNA gyrase inhibitor	2.59	0.004
g2903.t1	W817_11660	*sbcB*	exonuclease I	2.1	0.01
g2926.t1	W817_11805	*wcaL*	colanic acid biosynthesis glycosyltransferase WcaL	2.35	0.036
g2927.t1	W817_11810	*wcaK*	colanic acid biosynthesis protein	2.51	0.007
g2928.t1	W817_11815	*wzxC*	colanic acid exporter	3.4	0.02
g2932.t1	W817_11840	*wcaI*	glycosyl transferase	3.7	0.045
g2933.t1	W817_11845	*wcaH*	GDP-mannose mannosyl hydrolase NudD	4.46	0.001
g2934.t1	W817_11850	*wcaG*	GDP-fucose synthetase	5.63	0.035
g2935.t1	W817_11855	*gmd*	GDP-mannose 4,6-dehydratase	7.8	0.041
g2936.t1	W817_11860	*wcaF*	acyl transferase	3.76	0.015
g2937.t1	W817_11865	*wcaE*	glycosyl transferase	4.12	0.012
g294.t1	W817_23535	*fxsA*	exclusion suppressor FxsA	1.46	0.005
g2941.t1	W817_11875	*wcaC*	colanic acid biosynthesis glycosyltransferase WcaC	5.48	0.002
g2943.t1	W817_11885	*wcaA*	glycosyl transferase	6.59	0.016
g2944.t1	W817_11890	*wzc*	tyrosine kinase	3.49	0.036
g2945.t1	W817_11895	*wzb*	protein-tyrosine phosphatase	12.24	0.024
g2946.t1	W817_11900	*wza*	polysaccharide export protein Wza	9.12	0.015
g2954.t1	W817_11935	*yegE*	predicted diguanylate cyclase yegE	1.43	0.009
g2965.t1	W817_11980	*mdtB*	MdtABC-TolC efflux pump	1.15	0.002
g2966.t1	W817_11985	*mdtC*	MdtABC-TolC efflux pump	1.52	0.026
g2976.t1	W817_12025	*C2359*	hypothetical protein	0.16	0.025
g298.t1	W817_23555	*yjeI*	membrane protein	2.13	0.009
g2995.t1	W817_12115	*thiM*	hydroxyethylthiazole kinase	0.38	0.046
g2997.t1	W817_12125	*yohM*	cobalt transporter	0.5	0.037
g3000.t1	W817_12135	*yehA*	fimbrial protein	0.21	0.002
g3001.t1	W817_12140	*yehB*	fimbrial outer membrane usher protein	0.09	0.025
g3002.t1	W817_12145	*yehC*	fimbrial assembly chaperone protein StcB	0.22	0.001
g3016.t1	W817_12205	*yehR*	hypothetical lipoprotein	0.42	0.009
g302.t1	W817_23575	*ecnA*	entericidin A	0.3	0.011
g3029.t1	W817_12270	*yohC*	membrane protein	2.38	0.019
g3051.t1	W817_12370	*cirA*	catecholate siderophore receptor CirA	0.28	0.019
g3066.t1	W817_12435	*C2444*	PTS fructose transporter subunit IIA	0.48	0.02
g3083.t1	W817_12510	*yejG*	hypothetical protein	0.38	0.025
g3093.t1	W817_12560	*narP*	nitrate/nitrite response regulator NarP	1.38	0.024
g3102.t1	W817_12595	*ccmB*	Heme exporter protein CcmB	2.72	0.005
g3103.t1	W817_12600	*ccmA*	cytochrome C biogenesis protein CcmA	2.12	0.023
g3113.t1	W817_12645	*mqo*	malate:quinone oxidoreductase	0.36	0.008
g3120.t1	W817_12670	*ompC*	outer membrane porin protein C	3.29	0.031
g3122.t1	W817_12675	*yojN*	RcsD phosphotransferase	0.09	0.027
g3127.t1	W817_12690	*atoS*	histidine kinase	2.18	0.036
g3137.t1	W817_12735	*yfaT*	hypothetical protein	0.35	0.028
g3150.t1	W817_12785	*glpT*	sugar phosphate permease	0.5	0.031
g3160.t1	W817_12820	*yfaU*	2-keto-3-deoxy-L-rhamnonate aldolase	0.45	0.03
g3222.t1	W817_13100	*folX*	dihydroneopterin triphosphate epimerase	2.5	0.017
g3229.t1	W817_13135	*argT*	Lysine-arginine-ornithine-binding periplasmic protein	2.58	0.019
g3243.t1	W817_13200	*fabB*	3-oxoacyl-ACP synthase	2.15	0.015
g3259.t1	W817_13275	*yfcV*	fimbrial yfcV	0.42	0.016
g3268.t1	W817_13325	*intC*	putative prophage CPS-53 integrase	0.31	0.042
g327.t1	W817_23705	*hfq*	RNA-binding protein Hfq	1.92	0.034
g3287.t1	W817_13440	*hkbM*	hypothetical protein	5.48	0.018
g3290.t1	W817_13460	*C2660*	lysozyme	0.48	0.048
g3301.t1	W817_13550	*C2677*	repressor	2.6	0.001

g3315.t1	W817_13665	*emrY*	multidrug resistance protein Y	0.21	0.043
g3317.t1	W817_13670	*emrK*	EmrKY-TolC multidrug resistance efflux pump	0.15	0.01
g3321.t1	W817_13690	*yfdV*	auxin efflux transporter type protein	0.21	0.012
g3323.t1	W817_13700	*yfdW*	formyl-CoA transferase	0.3	0.028
g3324.t1	W817_13705	*yfdX*	hypothetical protein	0.05	0.001
g3365.t1	W817_13895	*cysM*	cysteine synthase	2.59	0.004
g3378.t1	W817_13940	*cysU*	sulfate/thiosulfate transporter	4.33	0.039
g3389.t1	W817_13980	*ypeA*	acetyltransferase	2.22	0.025
g3390.t1	W817_13985	*amiA*	N-acetylmuramoyl-l-alanine amidase I	0.49	0.022
g340.t1	W817_23760	*yjfK*	hypothetical protein	0.35	0.039
g3407.t1	W817_14060	*eutT*	ethanolamine utilization cobalamin adenosyltransferase	2.27	0.042
g3408.t1	W817_14065	*eutQ*	ethanolamine utilization protein EutQ	2.4	0.024
g342.t1	W817_23770	*yjfM*	membrane protein	0.47	0.039
g3440.t1	W817_14200	*upp*	uracil phosphoribosyltransferase	0.46	0.021
g3447.t1	W817_14225	*yfgF*	cyclic di-GMP phosphodiesterase	0.35	0.014
g3449.t1	W817_14240	*yfgH*	membrane protein	0.2	0.028
g3459.t1	W817_14275	*C2830*	intimin	0.24	0.048
g346.t1	W817_23785	*yjfN*	hypothetical protein	2.68	0.006
g3472.t1	W817_14335	*sseA*	3-mercaptopyruvate sulfurtransferase	2.54	0.019
g3524.t1	W817_14565	*rseA*	anti-RNA polymerase sigma factor σE	1.53	0.013
g3532.t1	W817_14610	*ung*	uracil-DNA glycosylase	2.19	0.023
g3537.t1	W817_14630	*yfiP*	hypothetical protein	0.29	0.004
g3541.t1	W817_14650	*kgtP*	alpha-ketoglutarate permease	2.13	0.009
g3545.t1	W817_14665	*insK*	IS150 conserved protein InsB	1.84	0.018
g3551.t1	W817_14725	*C2931*	translation inhibitor protein RaiA	2.75	0.033
g3557.t1	W817_14755	*yfiN*	predicted diguanylate cyclase yfiN	0.64	0.03
g3569.t1	W817_14810	*rceN*	DNA repair protein RecN	0.34	0.018
g3570.t1	W817_14815	*bamE*	outer membrane protein assembly factor	1.74	0.033
g3577.t1	W817_14885	*ycfK*	integrase	0.42	0.043
g3604.t1	W817_15000	*C2987*	endonuclease	0.06	0.036
g3623.t1	W817_15115	*C3009*	integrase	0.23	0.018
g363.t1	W817_23870	*C4805*	toxin HipA	0.48	0.024
g3638.t1	W817_15190	*stpA*	DNA-binding protein StpA	0.38	0.017
g364.t1	W817_23875	*exuT*	hexuronate transporter ExuT	0.2	0.03
g3648.t1	W817_15235	*nrdF*	ribonucleotide-diphosphate reductase subunit beta	0.41	0.039
g3652.t1	W817_15255	*ygaY*	transporter	0.18	0.012
g3668.t1	W817_15335	*csrA*	carbon storage regulator	1.72	0.035
g3669.t1	W817_15340	*alaS*	alanyl-tRNA synthetase	1.68	0.028
g3713.t1	W817_15555	*pphB*	serine/threonine protein phosphatase	0.46	0.047
g3722.t1	W817_15600	*rpoS*	RNA polymerase sigma factor RpoS	1.76	0.001
g3731.t1	W817_15640	*ygbE*		2.15	0.041
g3733.t1	W817_15650	*cysN*	sulfate adenylyltransferase subunit 1	2.48	0.016
g3736.t1	W817_15655	*cysD*	sulfate adenylyltransferase subunit 2	3.99	0.003
g3756.t1	W817_15750	*C3143*	membrane protein	0.38	0.003
g3768.t1	W817_15805	*gudD*	glucarate dehydratase	3.41	0.04
g3770.t1	W817_15810	*ygcY*	glucarate dehydratase	2.19	0.011
g3777.t1	W817_15850	*ygdH*	LOG family protein ygdH	2.27	0.007
g3782.t1	W817_15875	*fucA*	fuculose phosphate aldolase	2.29	0.03
g3786.t1	W817_15890	*fucK*	L-fuculokinase	2.55	0.048
g3788.t1	W817_15900	*fucR*	L-fucose operon activator	2.1	0.04
g3798.t1	W817_15960	*C3190*	hypothetical protein	0.47	0.029
g3799.t1	W817_15965	*C3191*	EvpB/family type VI secretion protein	0.37	0.042
g3813.t1	W817_16015	*C3203*	type VI secretion protein VasK	0.46	0.013
g3814.t1	W817_16020	*C3204*	Uncharacterized protein ImpA	0.33	0.049
g3815.t1		*C3205*	type VI secretion system protein TssA	0.1	0.047
g3822.t1	W817_16075	*C3212*	phosphoglycerate dehydrogenase	0.44	0.043
g3824.t1	W817_16085	*C3214*	beta-cystathionase	0.29	0.041
g3836.t1	W817_16145	*ppdA*	prepilin peptidase dependent protein A	4.15	0.014
g3837.t1	W817_16150	*C3229*	thymidylate synthase	3.46	0.023
g384.t1	W817_23955	*ytfJ*	hypothetical protein	2.07	0.002
g3844.t1	W817_16180	*ygdR*	hypothetical protein	2.95	0.016
g385.t1	W817_23960	*ytfK*	hypothetical protein	4.05	0.029
g3850.t1	W817_16210	*lysR*	transcriptional regulator	0.41	0.016
g3854.t1	W817_16230	*kduI*	5-keto-4-deoxyuronate isomerase	0.49	0.004

g3857.t1	W817_16240	*C3248*	septum formation protein	0.15	0.026
g3862.t1	W817_16270	*ygeV*	Fis family transcriptional regulator	2.9	0.005
g387.t1	W817_23970	*msrA*	methionine sulfoxide reductase A	2.11	0.021
g3871.t1	W817_16310	*ygfJ*	Molybdenum cofactor cytidylyltransferase	0.19	0.038
g3893.t1	W817_16425	*ygfZ*	global regulator	2.2	0.04
g3895.t1	W817_16435	*yqfB*	hypothetical protein	2.12	0.001
g3902.t1	W817_16470	*pepP*	Xaa-Pro aminopeptidase	2.23	0.003
g3905.t1	W817_16485	*ygfA*	5-formyltetrahydrofolate cyclo-ligase	0.21	0.013
g3912.t1	W817_16525	*yggE*	oxidative stress defense protein	1.44	0.016
g3928.t1	W817_16595	*C3319*	L-sorbose 1-phosphate reductase	0.3	0.001
g3934.t1	W817_16620	*yggG*	metallopeptidase	2.08	0.001
g3954.t1	W817_16715	*yggW*	HemN family oxidoreductase	0.46	0.04
g3996.t1	W817_16935	*glcG*	hypothetical protein	0.29	0.03
g3998.t1	W817_16940	*glcF*	glycolate oxidase	0.33	0.037
g3999.t1	W817_16945	*glcE*	FAD-binding protein	0.38	0.004
g4001.t1	W817_16955	*glcC*	GntR family transcriptional regulator	3.45	0.008
g4004.t1	W817_16965	*C3397*	acyl-CoA synthetase	0.37	0.011
g4005.t1	W817_16970	*C3398*	short-chain dehydrogenase	0.15	0.023
g401.t1	W817_24030	*pmbA*	peptidase PmbA	1.8	0.003
g4013.t1	W817_17010	*yghR*	hypothetical protein	0.42	0.042
g4014.t1	W817_17015	*yghS*	hypothetical protein	0.33	0.035
g4029.t1	W817_17090	*yghZ*	L-glyceraldehyde 3-phosphate reductase	2.4	0.014
g403.t1	W817_24035	*cybC*	cytochrome b562	2.52	0.017
g4039.t1	W817_17135	*yqhD*	alcohol dehydrogenase YqhD	2.95	0.02
g4042.t1	W817_17150	*yqhH*	lipoprotein YqhH	1.33	0.047
g4050.t1	W817_17190	*ygiK*	c4-dicarboxylate permease	0.51	0.021
g4058.t1	W817_17225"	*qseB*	quorum sensing two-component response regulator	1.5	0.018
g4061.t1	W817_17255	*ygiN*	quinol monooxygenase	2.67	0.022
g4075.t1	W817_17315	*tolC*	outer membrane channel protein	1.5	0.001
g4076.t1	W817_17320	*ygiB*	membrane protein	2.18	0.048
g4082.t1	W817_17340	*C3475*	disulfide oxidoreductase	2.2	0.025
g4086.t1	W817_17360	*ygiL*	putative Yqi fimbriae subunit YgiL precursor	0.35	0.004
g4092.t1	W817_17390	*glgS*	glycogen synthesis protein GlgS	4.18	0.021
g4093.t1	W817_17395	*yqiJ*	membrane protein	0.14	0.033
g4094.t1	W817_17400	*yqiK*	membrane protein	0.16	0.022
g4104.t1	W817_17450	*ygiP*	LysR family transcriptional regulator	0.49	0.012
g4121.t1	W817_17540	*ygjI*	amino acid permease	0.19	0.015
g4128.t1	W817_17575	*ygjR*	oxidoreductase	2.34	0.003
g4133.t1	W817_17595	*uxaA*	altronate hydrolase	2.68	0.042
g4135.t1		*exuT*	hexuronate transporter	1.8	0.013
g4154.t1	W817_17700	*tdcD*	propionate kinase	0.45	0.017
g4172.t1	W817_17795	*agaY*	tagatose-bisphosphate aldolase 2	0.23	0.033
g4175.t1	W817_17810	*agaD*	PTS N-acetylgalactosamine transporter subunit IID	2.68	0.025
g4185.t1	W817_17855	*yhbO*	general stress protein	0.49	0.039
g4197.t1	W817_17900	*deaD*	Cold-shock DEAD-box protein A	0.42	0.013
g421.t1	W817_24115	*yjgK*	Toxin-antitoxin biofilm protein TabA	2.18	0.031
g4239.t1	W817_18100	*rpoN*	RNA polymerase factor sigma-54	2.46	0.016
g4241.t1	W817_18105	*hpf*	ribosome hibernation promoting factor HPF	2.66	0.041
g4256.t1	W817_18175	*YhcJ*	N-acetylmannosamine-6-phosphate 2-epimerase	2.16	0.007
g4257.t1	W817_18180	*nanT*	sialic acid transporter	3.16	0.019
g4265.t1	W817_18220	*yhcB*	hypothetical protein	2.18	0.002
g4266.t1	W817_18225	*degQ*	serine endoprotease DegQ	1.55	0.029
g4272.t1	W817_18250	*yhcO*	hypothetical protein	0.25	0.028
g4273.t1	W817_18255	*C3671*	hydroxybenzoic acid transporter	0.3	0.037
g4277.t1	W817_18275	*tldD*	csrA activity inhibitor TldD	1.75	0.024
g4280.t1	W817_18290	*Maf*	maf-like protein YhdE	0.28	0.021
g4281.t1	W817_18295	*mreD*	rod shape-determining protein MreD	0.22	0.049
g4282.t1	W817_18300	*mreC*	rod shape-determining protein MreC	0.26	0.024
g4285.t1	W817_18315	*yhdH*	quinone oxidoreductase	2.37	0.048
g4293.t1	W817_18345	*rbsP*	D-ribose transporter ATP binding protein	0.21	0.038
g4297.t1	W817_18365	*C3698*	sugar kinase	2.22	0.012
g4308.t1	W817_18425	*yhdV*	hypothetical protein YhdV	2.45	0.003
g4313.t1	W817_18445	*artP*	arginine ABC transporter ATP-binding protein	2.41	0.012
g4325.t1	W817_18535	*trkA*	potassium transporter peripheral membrane protein	0.39	0.013

g4326.t1	W817_18540	*mscL*	large-conductance mechanosensitive channel	2.16	0.018
g436.t1	W817_24175	*idnT*	fructuronate transporter	0.68	0.021
g4371.t1	W817_18760	*pshM*	general secretion pathway protein M	0.37	0.019
g4385.t1	W817_18825	*fkpA*	periplasmic peptidylprolyl cis,trans isomerase	1.23	0.004
g4399.t1	W817_18880	*crp*	CRP transcriptional dual regulator	1.91	0.037
g4402.t1	W817_18890	*argD*	succinyldiaminopimelate aminotransferase	0.43	0.016
g4405.t1	W817_18910	*ppiA*	peptidylprolyl-cis-trans-isomerase A	1.16	0.012
g4412.t1	W817_18940	*yhfL*	membrane protein	0.51	0.001
g4421.t1	W817_18980	*yhfZ*	hypothetical protein	2.57	0.003
g443.t1	W817_24215	*yjgX*	sulfatase	0.26	0.013
g4434.t1	W817_19040	*mrcA*	penicillin-binding protein 1A	0.51	0.014
g4438.t1	W817_19055	*igaA*	intracellular growth attenuator protein igaA	0.75	0.007
g444.t1	W817_24220	*hek*	adhesin/virulence factor Hek	0.1	0.013
g4456.t1	W817_19120	*yhgA*	transposase	2.67	0.019
g4459.t1	W817_19140	*nfuA*	iron-sulfur cluster scaffold protein	1.85	0.033
g447.t1	W817_24240	*papG*	p pilus adhesin PapG protein	0.12	0.033
g4473.t1	W817_19205	*C3928*	hypothetical protein	0.03	0.04
g4475.t1	W817_19215	*sfmH*	hypothetical protein	0.13	0.045
g4477.t1	W817_19235	*aufC*	outer membrane usher protein	0.17	0.038
g448.t1	W817_24245	*papF*	fimbrial adapter papF	0.12	0.019
g4483.t1	W817_19255	*glgA*	glycogen synthase	2.64	0.034
g4485.t1	W817_19265	*glgX*	glycogen-debranching protein	2.86	0.04
g4494.t1	W817_19305	*yhhX*	oxidoreductase	2.11	0.011
g4498.t1	W817_19335	*yhhA*	membrane protein	2.58	0.049
g450.t1	W817_24255	*papK*	fimbrial adapter PapK	0.24	0.027
g4516.t1	W817_19415	*C3973*	PTS fructose transporter subunit IID	0.12	0.042
g4518.t1	W817_19425	*C3975*	dihydrodipicolinate synthase	0.15	0.014
g4542.t1	W817_19540	*mngR*	regulatory protein	2.2	0.014
g4551.t1	W817_19585	*yhiI*	membrane protein	2.14	0.018
g4556.t1	W817_19610	*uspA*	universal stress protein A	2.67	0.032
g4557.t1	W817_19615	*yhiP*	MFS transporter	1.78	0.048
g4567.t1	W817_19660	*yhiF*	LuxR family transcriptional regulator	0.29	0.014
g457.t1	W817_24335	*C4905*	F17 fimbrial usher	0.17	0.05
g4572.t1	W817_19680	*chuW*	coproporphyrinogen III oxidase	0.49	0.011
g4582.t1	W817_19730	*yhiU*	multidrug transporter	0.33	0.015
g4584.t1	W817_19735	*yhiV*	multidrug transporter	0.5	0.012
g4596.t1	W817_19795	*kdgK*	ketodeoxygluconokinase	2.24	0.003
g4656.t1	W817_20070	*yiaK*	2,3-diketo-L-gulonate reductase	2.66	0.03
g4668.t1	W817_20130	*yiaY*	alcohol dehydrogenase	0.38	0.02
g4692.t1	W817_20230	*secB*	preprotein translocase subunit SecB	1.69	0.001
g4699.t1	W817_20260	*yibD*	glycosyl transferase	0.42	0.046
g4701.t1	W817_20270	*kbl*	2-amino-3-ketobutyrate CoA ligase	2.14	0.041
g4702.t1	W817_20275	*rfaD*	ADP-L-glycero-D-mannoheptose-6-epimerase	1.33	0.003
g4707.t1	W817_20305	*rfaY*	lipopolysaccharide core heptose(II) kinase RfaY	1.52	0.046
g4720.t1	W817_20360	*radC*	DNA repair protein	2.86	0.001
g4723.t1	W817_20375	*slmA*	division inhibitor protein	0.48	0.004
g4735.t1	W817_20430	*recG*	ATP-dependent DNA helicase RecG	0.51	0.024
g4736.t1	W817_20435	*C4198*	sodium/glutamate symport carrier protein GltS	2.35	0.006
g4752.t1	W817_20505	*rhuM*	2-hydroxyacid dehydrogenase	0.52	0.007
g4764.t1	W817_20570	*uhpA*	DNA-binding transcriptional activator	0.5	0.001
g4768.t1	W817_20590	*yidF*	hypothetical protein	2.39	0.02
g477.t1	W817_24455	*C4929*	molybdopterin binding oxidoreductase	0.27	0.018
g4796.t1	W817_20740	*tnaA*	L-cysteine desulfhydrase	0.39	0.024
g4797.t1	W817_20745	*tnaB*	tryptophan permease	0.25	0.012
g4798.t1	W817_20750	*yidY*	multidrug transporter	0.49	0.026
g4803.t1	W817_20775	*yieH*	6-phosphogluconate phosphatase	0.38	0.011
g481.t1	W817_24470	*C4932*	sensor histidine kinase	0.33	0.049
g4812.t1	W817_20815	*phoU*	phosphate transport system protein PhoU	2.42	0.03
g4813.t1	W817_20820	*pstB*	phosphate ABC transporter ATP-binding protein	2.34	0.008
g4815.t1	W817_20830	*pstC*	phosphate transporter permease subunit PstC	2.4	0.032
g4830.t1	W817_20905	*gidA*	glucose-inhibited cell division protein A	0.47	0.036
g484.t1	W817_24500	*C4940*	hypothetical protein	0.06	0.044
g4841.t1	W817_20955	*rbsB*	D-ribose transporter subunit RbsB	2.25	0.005
g4846.t1	W817_21010	*hdfR*	HNS-dependent flhDC regulator	2.04	0.009

g4847.t1	W817_21015	*yifE*	hypothetical protein	2.64	0.002
g485.t1	W817_24505	*C4941*	hypothetical protein	0.07	0.049
g486.t1	W817_24510	*C4942*	hypothetical protein	0.22	0.026
g4869.t1	W817_21090	*rho*	transcription termination factor Rho	0.51	0.003
g49.t1	W817_00615	*traD*	conjugal transfer protein TraD	0.67	0.005
g4905.t1	W817_21255	*xerC*	tyrosine recombinase XerC	0.46	0.032
g4935.t1	W817_21425	*C4407*	FdrA	0.39	0.039
g494.t1	W817_24550	*C4952*	transcriptional regulator	0.06	0.037
g4942.t1	W817_21455	*C4412*	C4-dicarboxylate ABC transporter permease	2.53	0.031
g495.t1	W817_24555	*C4953*	hypothetical protein	0.17	0.012
g4994.t1	W817_21720	*yihX*	alpha-D-glucose-1-phosphatase	2.38	0.046
g5005.t1	W817_21785	*C4481*	sulfate ABC transporter substrate-binding protein	2.17	0.007
g5008.t1	W817_21795		hypothetical protein	0.28	0.031
g5032.t1	W817_21915	*yiiS*	DUF406 domain-containing protein YiiS	2.42	0.033
g5033.t1	W817_21920	*uspD*	universal stress protein D	2.5	0.025
g5040.t1	W817_21945	*yiiU*	septal ring assembly protein ZapB	2.09	0.006
g5041.t1	W817_21950	*menG*	ribonuclease activity regulator protein RraA	2.59	0.04
g5049.t1	W817_21990	*yiiX*	hypothetical protein	0.27	0.032
g5057.t1	W817_22020	*C4528*	5'-nucleotidase	0.44	0.025
g5060.t1	W817_22040	*metF*	5,10-methylenetetrahydrofolate reductase	0.23	0.023
g5063.t1	W817_22055	*yijF*	hypothetical protein	0.35	0.013
g5064.t1	W817_22065	*gldA*	glycerol dehydrogenase	3.96	0.041
g5066.t1	W817_22070	*talC*	fructose-bisphosphate aldolase	3.07	0.035
g5072.t1	W817_22090	*pflD*	formate acetyltransferase	2.85	0.018
g5073.t1	W817_22095	*pflC*	pyruvate formate lyase II activase	3.08	0.005
g5076.t1	W817_22110	*yijP*	membrane protein	0.49	0.02
g5084.t1	W817_22145	*yjiZ*	L-galactonate transporter	0.25	0.021
g5089.t1	W817_22170	*fabR*	transcriptional regulator	1.92	0.044
g5090.t1	W817_22175	*yijD*	membrane protein	2.49	0.005
g5116.t1	W817_22325	*rsd*	anti-RNA polymerase sigma 70 factor	2.31	0.002
g5130.t1	W817_22405	*yjaA*	stress response protein	0.37	0.046
g5137.t1	W817_22430	*aceK*	isocitrate dehydrogenase	2.2	0.001
g5156.t1	W817_22525	*yjbE*	exopolysaccharide production protein	12.37	0.035
g5158.t1	W817_22535	*yjbG*	hypothetical protein	2.96	0.032
g5168.t1	W817_22595	*ubiC*	chorismate pyruvate lyase	2.41	0.004
g5170.t1	W817_22605	*plsB*	glycerol-3-phosphate O-acyltransferase	1.34	0.027
g518.t1	W817_24660	*C4973*	transcriptional regulator	0.46	0.004
g523.t1	W817_24685	*C4978*	sodium:proton antiporter	0.49	0.008
g524.t1	W817_24690	*C4979*	transposase IS2	0.48	0.024
g527.t1	W817_24720	*C4984*	GTPase	0.12	0.039
g538.t1	W817_24770	*yeeV2*	toxin	0.16	0.04
g544.t1	W817_24805	*C5006*	N-acetylneuraminic acid mutarotase	0.49	0.016
g548.t1	W817_24835	*fimI*	fimbrin fimI	0.39	0.008
g549.t1	W817_24840	*fimC*	molecular chaperone FimC	0.31	0.014
g55.t1	W817_00645	*P144*	endonuclease	0.42	0.004
g550.t1	W817_24845	*fimD*	fimbrial protein FimD	0.32	0.016
g556.t1	W817_24860	*fimH*	type 1 fimbiral adhesin FimH	0.53	0.004
g561.t1	W817_24890	*iraD*	DNA replication protein	0.43	0.034
g575.t1	W817_24955	*igbR*	transcriptional regulator DhaR	0.07	0.043
g576.t1	W817_24960	*ibeA*	invasion protein	0.35	0.032
g578.t1	W817_24970	*yjiE*	cell density-dependent motility repressor	0.26	0.004
g586.t1	W817_25020	*yjiL*	hypothetical protein	0.31	0.011
g596.t1	W817_25065	*hsdR*	endonuclease R	2.96	0.008
g601.t1	W817_25085	*yjiY*	carbon starvation protein CstA	2.19	0.027
g608.t1	W817_25120	*yjjN*	galactonate oxidoreductase	0.27	0.027
g616.t1	W817_25155	*yjjQ*	LuxR family transcriptional regulator	0.14	0.012
g620.t1	W817_25195	*rsmC*	16S rRNA methyltransferase	0.84	0.024
g627.t1	W817_25215	*prfC*	peptide chain release factor 3	0.43	0.028
g648.t1	W817_25320	*slt*	soluble lytic murein transglycosylase	1.49	0.02
g649.t1	W817_25325	*trpR*	Trp operon repressor	4.38	0.032
g65.t1	W817_00045	*P012*	membrane protein	0.36	0.009
g653.t1	W817_25345	*creA*	hypothetical protein	1.54	0.031
g67.t1	W817_00050	*P013*	ABC transporter permease	0.29	0.02
g673.t1	W817_00710	*C0019*	hypothetical protein	0.27	0.003

g686.t1	W817_00770	*rihA*	ribonucleoside hydrolase	2.17	0.019
g689.t1	W817_00785	*carA*	carbamoyl phosphate synthase small subunit	0.33	0.016
g693.t1	W817_00805	*caiE*	carnitine operon protein CaiE	0.41	0.002
g695.t1	W817_00815	*caiC*	crotonobetaine/carnitine-CoA ligase	0.48	0.044
g696.t1	W817_00820	*caiB*	crotonobetainyl-CoA:carnitine CoA-transferase	0.42	0.038
g698.t1	W817_00830	*caiT*	antiporter	0.32	0.007
g714.t1	W817_00910	*lptD*	LPS assembly outer membrane complex protein	1.35	0.041
g72.t1	W817_00075	*yihX*	glucose-1-phosphatase	3.12	0.003
g725.t1	W817_00970	*thiQ*	thiamine ABC transporter ATP-binding protein	0.37	0.008
g726.t1	W817_00975	*thiP*	thiamine transporter membrane protein	0.44	0.014
g737.t1	W817_01030	*fruR*	DNA-binding transcriptional dual regulator	1.61	0.006
g738.t1	W817_01035	*mraZ*	cell division protein MraZ	2.18	0.009
g742.t1	W817_01050	*ftsI*	cell division protein FtsI	2.56	0.028
g744.t1	W817_01055	*murE*	peptide ligase	2.83	0.002
g755.t1	W817_01110	*lpxC*	UDP-3-O-acyl-N-acetylglucosamine deacetylase	1.26	0.004
g764.t1	W817_01150	*hofC*	type IV pilin biogenesis protein	0.42	0.002
g771.t1	W817_01185	*usp*	HNH nuclease	0.38	0.002
g784.t1	W817_01245	*yacL*	hypothetical protein	2.24	0.043
g786.t1	W817_01255	*speE*	spermidine synthase	1.76	0.029
g79.t1	W817_00145	*P031*	transposase ISPsy3	0.31	0.02
g800.t1	W817_01320	*panB*	3-methyl-2-oxobutanoate hydroxymethyltransferase	2.22	0.03
g802.t1	W817_01330	*yadK*	fimbrial protein	0.22	0.033
g806.t1	W817_01350	*ecpD*	molecular chaperone EcpD	0.5	0.022
g81.t1	W817_00135	*cjrC*	colicin Js sensitive receptor protein	0.42	0.015
g812.t1	W817_01380	*sfsA*	sugar fermentation stimulation protein A	2.58	0.012
g818.t1	W817_01410	*fhuC*	ferrichrome transport ATP-binding protein FhuC	0.46	0.025
g821.t1	W817_01420	*fhuB*	ferrichrome transport system permease protein fhuB	0.31	0.04
g826.t1	W817_01435	*yadR*	iron-sulfur cluster insertion protein ErpA	2.13	0.012
g83.t1	W817_00125	*cjrA*	putative inner membrane lipoprotein	0.52	0.039
g833.t1	W817_01470	*yaeH*	hypothetical protein	2.21	0.01
g838.t1	W817_01485	*map*	methionine aminopeptidase	2.14	0.046
g852.t1	W817_01545	*fabZ*	3R-hydroxymyristoyl ACP dehydratase	1.79	0.008
g860.t1	W817_01575	*ldcC*	lysine decarboxylase CadA	1.36	0.023
g864.t1	W817_01595	*yaeP*	hypothetical protein	2.16	0.017
g869.t1	W817_01615	*yaeF*	hypothetical protein	0.41	0.041
g889.t1	W817_01740	*C0237*	Hcp	0.43	0.003
g912.t1	W817_01855	*C0258*	conjugative transfer protein	0.25	0.044
g914.t1	W817_01865	*ykfE*	C-lysozyme inhibitor	2.54	0.045
g919.t1	W817_01890	*yafL*	endopeptidase	0.49	0.046
g921.t1	W817_01900	*flhA*	flagellar biosynthesis protein FlhA	0.22	0.042
g927.t1	W817_01930	*C0275*	hypothetical protein	0.43	0.002
g933.t1	W817_01955	*crl*	transcriptional regulator	2.1	0.002
g939.t1	W817_01990	*hbp*	hemoglobin-binding protease hbp autotransporter	0.52	0.002
g946.t1	W817_02035	*yagX*	CFA/I fimbrial subunit C usher protein	0.36	0.005
g975.t1	W817_02185	*betB*	betaine-aldehyde dehydrogenase	2.05	0.008
g978.t1	W817_02200	*yahA*	cyclic di-GMP phosphodiesterase	0.53	0.045
g999.t1	W817_02290	*atoC*	acetoacetate metabolism regulatory protein AtoC	2.41	0.011
