# Supplementary figures and images for: The role of the bacterial protease Prc in the uropathogenesis of extraintestinal pathogenic Escherichia coli
Source: J Biomed Sci. 2020 Jan 3;27:14. doi: 10.1186/s12929-019-0605-y (PMC6941253; doi:10.1186/s12929-019-0605-y)

**a**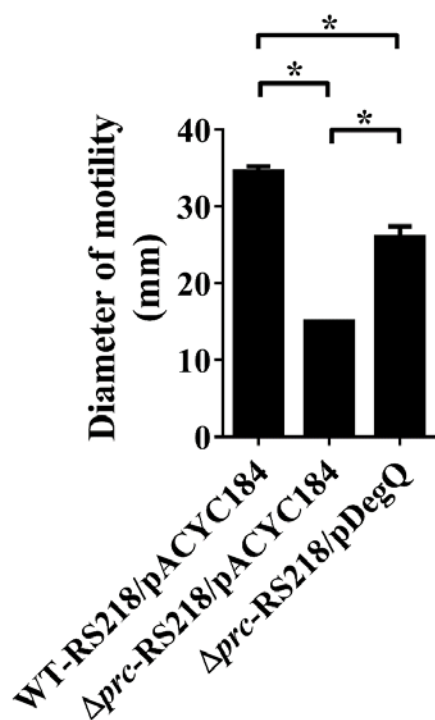**b**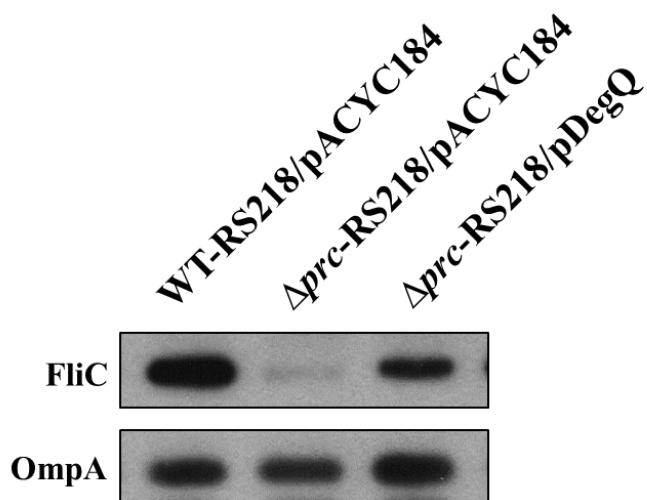**c**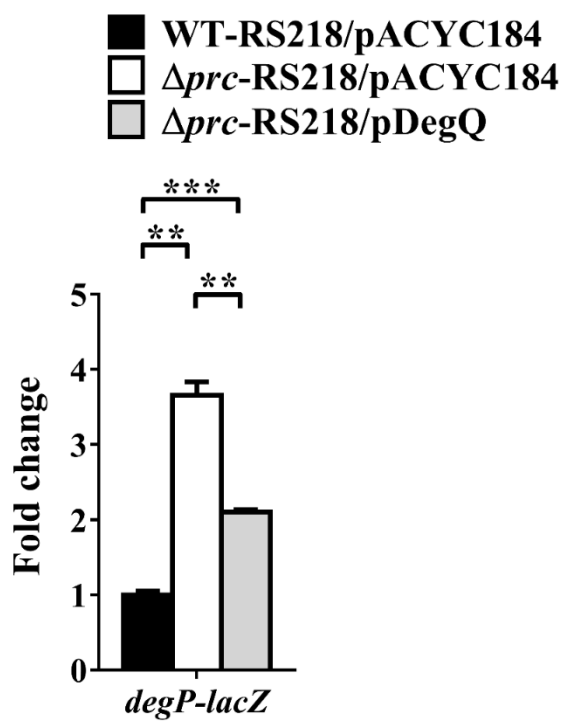

Supplement: Supplementary file 4 — Additional file 4: Figure S1. The effects of overexpression of DegQ on motility, FliC expression, and activation of σE signaling. (a) Motility diameter of WT-RS218/pACYC184, Δprc-RS218/pACYC184, and Δprc-RS218/pDegQ. (b) FliC levels in WT-RS218/pACYC184, Δprc-RS218/pACYC184, and Δprc-RS218/pDegQ. (c) Promoter activity of degP in WT-RS218/pACYC184, Δprc-RS218/pACYC184, and Δprc-RS218/pDegQ. degP is positively regulated by the σE signaling system. The promoter activity of degP can reflect the activation level of σE signaling [file 12929_2019_605_MOESM4_ESM.pdf]
